# Memory-Like Inflammatory Responses of Microglia to Rising Doses of LPS: Key Role of PI3Kγ

**DOI:** 10.3389/fimmu.2019.02492

**Published:** 2019-11-08

**Authors:** Trim Lajqi, Guang-Ping Lang, Fabienne Haas, David L. Williams, Hannes Hudalla, Michael Bauer, Marco Groth, Reinhard Wetzker, Reinhard Bauer

**Affiliations:** ^1^Institute of Molecular Cell Biology, Jena University Hospital, Jena, Germany; ^2^Department of Neonatology, Heidelberg University Children's Hospital, Heidelberg, Germany; ^3^Department of Surgery and Center of Excellence in Inflammation, Infectious Disease and Immunity, Quillen College of Medicine, East Tennessee State University, Johnson City, TN, United States; ^4^Department of Anesthesiology and Intensive Care Medicine, Jena University Hospital, Jena, Germany; ^5^Leibniz Institute on Aging–Fritz Lipmann Institute, CF DNA Sequencing, Jena, Germany

**Keywords:** microglia, PI3Kγ, LPS, β-glucan, training, tolerance, phagocytosis

## Abstract

Trained immunity and immune tolerance have been identified as long-term response patterns of the innate immune system. The causes of these opposing reactions remain elusive. Here, we report about differential inflammatory responses of microglial cells derived from neonatal mouse brain to increasing doses of the endotoxin LPS. Prolonged priming with ultra-low LPS doses provokes trained immunity, i.e., increased production of pro-inflammatory mediators in comparison to the unprimed control. In contrast, priming with high doses of LPS induces immune tolerance, implying decreased production of inflammatory mediators and pronounced release of anti-inflammatory cytokines. Investigation of the signaling processes and cell functions involved in these memory-like immune responses reveals the essential role of phosphoinositide 3-kinase γ (PI3Kγ), one of the phosphoinositide 3-kinase species highly expressed in innate immune cells. Together, our data suggest profound influence of preceding contacts with pathogens on the immune response of microglia. The impact of these interactions—trained immunity or immune tolerance—appears to be shaped by pathogen dose.

## Introduction

Innate immune cells act as first front line of the immune response to microbial pathogens. To this end, they exhibit an extraordinary sensitivity to recognize and respond on pathogen-associated molecular patterns (PAMPs). Endotoxins like bacterial lipopolysaccharides (LPS) have been shown to induce host responses to as few as 100 invading Gram-negative bacteria, corresponding to femtomoles of LPS (1–3). These highly sensitive reactions set off efficient elimination of the invading microorganisms.

Recent investigations move long-term adaptive responses of the innate immune system into focus. Specifically, it has been shown that innate immune cells acquire enhanced capability to respond to certain stimuli, a process termed trained immunity (4–6). Trained immunity is escorted by epigenetically fixed long-term reprogramming of intracellular signaling and metabolic pathways. In parallel, current mechanistic investigations reveal that the long-established tolerance response provoked by repeated LPS challenge is based on similar molecular mechanisms including changes in LPS signaling, epigenetic markers, and chromatin remodeling (7). The causes of these opposing long-term response patterns to PAMP-induced priming of innate immune cells remain obscure. Of note, various PAMPs have been assigned to induce either training (as β-glucan) or tolerance responses (as LPS) (8). Alternatively, dose-dependent priming responses have been depicted [reviewed in (9)]. The response patterns observed includes training effects of low doses and tolerance development at high doses of LPS.

Microglial cells mediate the innate immune response inside the central nervous system (CNS) (10). In addition, they fulfill supportive functions for maintenance of tissue homeostasis, tissue support, neuroplasticity, and neuroprotection on the basis of their sensitive response on detected alterations in their surveillant microenvironment (11). Infections or insults of brain tissue induce immediate inflammatory responses of affected microglial cells (12). The related microglial activation is accompanied by the secretion of cytokines, generation of reactive oxygen species (ROS), and phagocytic activities (13–16). Recent seminal reports revealed memory-like immune responses of microglia toward PAMPs. Based on *in vitro* studies, Schaafsma and coworkers discovered immune tolerance after priming of microglial cells with LPS. Mechanistic analysis revealed epigenetic modifications as essential mediators of the immune-suppressed phenotype (17). Another recent study disclosed that peripherally applied inflammatory stimuli induce acute immune training and tolerance in the brain and lead to differential epigenetic reprogramming of microglia (18).

Our present report extends and deepens the investigations of both related studies. Primary microglial cells from neonatal mice have been primed with a broad dose range of LPS, and inflammatory responses to a fixed dose of LPS were assayed after 6 days of culture. Whereas, immune tolerance was observed at high priming dose of LPS, our data reveal trained immunity in response to ultra-low priming doses of LPS. Mechanistic examination discloses the signaling protein PI3Kγ as an essential mediator of the trained immunity. The implications of dose-dependent priming effects for microglial functional patterns in the CNS will be discussed.

## Methods

### Animals

PI3Kγ knockout mice (PI3Kγ^−/−^) (19) and mice carrying a targeted mutation causing loss of lipid kinase activity (PI3Kγ^KD/KD^) (20) were on the C57BL/6J background for >10 generations. Age-matched C57BL/6 mice were used as controls (wild type). The animal procedures were performed according to the guidelines on the protection of animals used for scientific purposes. Experiments were approved by the Thuringian State Office for Food Safety and Consumer Protection. For breeding, animals were maintained at 12-h light and dark cycles with free access to food and water. To prepare cell culture of primary microglial cells for *in vitro* experiments, neonatal (P0–P3) wild type, PI3Kγ^−/−^, and PI3Kγ^KD/KD^ mice have been used.

### Isolation and Culture of Primary Microglial Cells

Newborn animals were sacrificed and brains were carefully removed and transferred in autoclaved PBS. After removal of the meninges and brain stem, the brains were minced and washed in dissection medium. Mixed cultures from newborn mice brains were prepared and microglial cells were enriched as described previously (21, 22). Microglial cultures were maintained in Dulbecco's Modified Eagle's Medium (SIGMA #06429, endotoxin tested) and 10% heat-inactivated fetal bovine serum (FBS, Sigma-Aldrich #F7524, endotoxin tested and sterile filtered) supplemented with 100 U/ml penicillin, 100 μg/ml streptomycin, and 2.5 μg/ml amphotericin B. After 14 days, adherent microglial cells were separated from astrocytes by adding PBS-EDTA solution and careful shaking. After harvesting, microglial cells were seeded in adherent well plates. Purity of microglia was in the range between 95 and 98%, as confirmed by Iba1 staining (data not shown).

### Microglial Cell Stimulation

**Figure 1:**
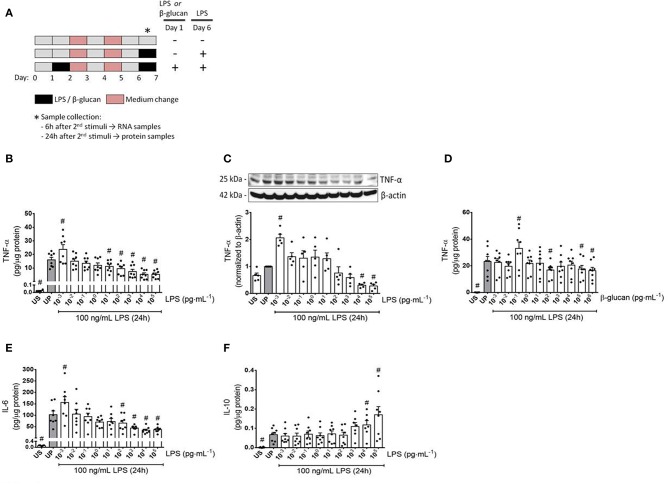
Cytokine responses of microglia primed with increasing doses of LPS and β-glucan. **(A)** Schematic view of the two-step approach to induce memory-like inflammatory responses. Primary microglial cells (75,000 cells/well) isolated from neonatal mice were seeded on day 0 and stimulated with increasing doses of LPS (1 fg/ml to 100 ng/ml) or β-glucan (1 fg/ml to 1 μg/ml) on day 1 for 24 h. On day 2, cells were washed up and followed by medium change on day 4. Thereafter, cells were left to rest and restimulated on day 6 with a fixed dose of LPS (100 ng/ml). Six h and 24 h after the second stimulation, samples have been collected and processed for further analysis. **(B–F)** Opposing inflammatory response of microglia after priming with increasing doses of LPS or β-glucan. Microglial cells were stimulated with increasing doses of LPS (**B,C**: 1 fg/ml to 100 ng/ml) or β-glucan (**D**: 1 fg/ml to 1 μg/ml) for 24 h and rechallenged on day 6 with a fixed dose of LPS (100 ng/ml). Supernatants and protein lysates have been collected 24 h after the second stimulus by LPS (100 ng/ml). Cytokine levels (**B,D**: TNF-α, *n* = 8; **E**: IL-6, *n* = 8; **F**: IL-10, *n* = 8) have been analyzed by ELISA (normalized to total protein concentration), whereas protein expression (C: TNF-α, *n* = 5) has been assayed by Western blotting and quantification (unprimed cells assigned as 1.0). Data are given as scatter dot plots, mean + SEM, ^#^*p* < 0.05, ^#^significant differences vs. unprimed condition (gray column). US, unstimulated; UP, unprimed.

In order to induce innate immune memory responses, microglial cells (75,000 cells/well) were seeded in a 12-well plate and treated according to the stimulation scheme depicted in Figure 1A. Cells were stimulated twice following a two-step procedure with an initial priming step using increasing doses of LPS (1 fg/ml to 100 ng/ml; *Escherichia coli* serotype 055:B5 obtained from Sigma-Aldrich, St. Louis, USA) or β-glucan (1 fg/ml to 1 μg/ml) for 24 h. Thereafter, cells were washed up and the medium was changed. A second medium change was performed on day 4. On day 6, microglial cells were restimulated with a fixed dose of LPS (100 ng/ml). In parallel, measurements were performed on unstimulated (US) cells as well as on unprimed (UP) cells (stimulation occurred solely on day 6 with a fixed dose of 100 ng/ml LPS).

### Antibodies

Monoclonal antibody against the catalytic subunit p110γ of PI3Kγ was produced in our laboratory (23). Other primary antibodies have been purchased from Cell Signaling (USA): tumor necrosis factor alpha (TNF-α) (#3707), phospho-NFκB p65 (S536) (#3033), phospho-Akt (Ser473) (#9271), Akt (#9272), phospho-p44/42 MAPK (ERK1/2) (Thr202/Tyr204) (#9106), p44/42 MAPK (ERK1/2) (#9107), MyD88 (#4283), and TLR4 (#14358).

Antibodies against inducible nitric oxide synthase (iNOS) was obtained from Abcam (UK, #ab3523) and against β-actin (#A2228 and #A5441) from Sigma-Aldrich (St. Louis, USA). Secondary HRP-coupled anti-rabbit and anti-mouse antibodies were purchased from KPL (Weden, Germany).

### SDS-PAGE Western Blotting

Cells were lysed using RIPA buffer containing 50 mM Tris–HCl, pH 8; 150 mM NaCl, 1% (v/v) NP-40, 0.5% (v/v) Na-deoxycholate, 0.1% (w/v) SDS, 100 mg/ml Pefa-Block, 1 mg/ml Pepstatin A, 10 mM sodium vanadate, and 1 mg/ml leupeptin. Samples were centrifuged (13,500 × *g* for 30 min at 4°C), and supernatants were mixed with 5×protein sample buffer (5% SDS, 33% glycerol, 25% β-mercaptoethanol, 83 mM Tris–HCl with pH to 6.8, and 0.1 mg/ml bromophenol blue) and heated for 5 min at 95°C. Protein samples were separated on 10% polyacrylamide gel, transferred to a 0.45-μm polyvinylidenfluoride (PVDF) membrane, and then immunoblotted with the above mentioned primary antibodies. Protein bands were detected by enhanced chemiluminescence reaction using LAS4000 camera (Fuji Photo Film Co., Tokyo, Japan). Quantification of the protein bands on the membrane was done using the FujiFilm Multi Gauge Ver. 3.0 software (Fuji Photo Film Co., Tokyo, Japan).

### Measurement of the Protein Concentration

Total protein concentration was determined using the Pierce™ 660 nm Protein Assay Kit (#22662) from Thermo Fisher Scientific (Massachusetts, USA). Ionic detergent compatibility reagent (IDCR) (#22663, Thermo Fischer Scientific) was used in order to increase the detergent compatibility and reduce interference. Absorbance was measured at 660 nm using a TECAN Infinite 200 Plate Reader (Tecan, Switzerland). Protein concentration has been calculated based on the values of the standard curve.

### Cytokine Measurement

Cytokine concentration in supernatants was measured using commercial enzyme-linked immunosorbent assay (ELISA) kits for TNF-α (#430902), IL-6 (#431302), and IL-10 (#431412) obtained from BioLegend (San Diego, CA). The absorbance was read at 450 nm with a second reference wavelength at 570 nm in VersaMax Microplate Reader (Molecular Devices, USA). Cytokine levels of TNF-α, IL-6, and IL-10 after the second stimulation were normalized against the protein concentrations of each sample and depicted as pg/μg of total protein.

### Gene Expression

Total RNA was extracted from microglial cells using QIAzol Lysis Reagent (#79306) purchased from Qiagen (Hilden, Germany). RNA concentration and quality were checked by using the Nanodrop ND-1000 machine (Peqlab, Erlangen, Germany). Complementary DNA (cDNA) was synthesized using RevertAid First Strand cDNA Synthesis kit (#K1612) from Thermo Fisher Scientific (Waltham, MA, USA). qPCR reaction was performed by using Realplex Mastercycler EpGradient S (Eppendorf AG, Germany). The following primer pairs were used: HIF-1α forward: CTCATCAGTTGCCACTTCC and HIF-1α reverse: TCATCTTCACTGTCTAGACCAC, MyD88 forward: TCCGGCAACTAGAACAGACAGACT and MyD88 reverse: GCGGCGACACCTTTTCTCAAT, TLR4 forward: ACCTGGCTGGTTTACACGTC and TLR4 reverse: CAGGCTGTTTGTTCCCAAAT, GAPDH forward: CATGGCCTTCCGTGTTTCCTA and GAPDH reverse: CCTGCTTCACCACCTTCTTGAT. GAPDH was used as housekeeping gene. Relative gene expression was calculated using the comparative C_T_ ($2_{\text{T}}^{-\Delta \Delta \text{C}}$) method (24).

### Measurement of ROS

ROS were measured using the H_2_DCFDA assay. The assay is based on the use of 2′,7′-dichlorodihydrofluorescein-diacetate (H2DCFDA; #D399, Thermo Fisher Scientific, Waltham, MA, USA), a membrane-permeable reduced form of fluorescein that reacts with ROS turning fluorescent. Therefore, microglial cells were seeded into white clear bottom 96-well plates (30,000 cells/well). After becoming adherent, cells were stimulated, as described above. For measurement, the medium was aspirated and 200 μl of H_2_DCFDA solution (stock 50 mM 1:1,000 in 10 mM HEPES/CaCl_2_) was added and cells were incubated for 20 min at 37°C. Thereafter, cells were carefully washed twice with a HEPES/CaCl_2_ solution. Measurement of intracellular ROS levels was performed at 485 nm excitation and 535 nm emission using a TECAN Infinite 200 Plate Reader (Tecan, Switzerland).

### *In vitro* Phagocytosis Assay

Phagocytic efficiency was investigated as described previously (25, 26). Microglial cells (75,000 cells/well) were seeded into cover slips in 12-well plates and prepared as described above. Fluorescein isothiocyanate (FITC)-labeled Zymosan A (*Saccharomyces cerevisiae*) Bio Particles (9,800 U/ml) (#Z2841, Thermo Fischer Scientific) were used to perform the phagocytosis assay. After 1 h incubation with Zymosan A at 37°C (5% CO_2_) cells were fixed with Formalin 5%, washed three times, and blocked with 10% NDS in PBS-Tween 0.1%. Cells were first incubated with Iba1 primary antibody (goat Iba1, #ab5076, Abcam) for 1 h at room temperature and thereafter washed with 1× PBS and incubated with secondary antibody (Alexa Fluor® 568 donkey anti goat IgG). Thereafter, cells were stained with DAPI solution for 10 min (1:1,000 in 1× PBS) and washed four times with PBS (1×). Cover slips were then mounted with Fluoromount-G (#0100-01, Southern Biotech) into microscope slides. Phagocytosed zymosan particles and cells of five independent visual fields were counted under a fluorescence microscope (Nikon Eclipse Ti, Nikon Instruments–Japan). The result of the phagocytosis was calculated by determining the phagocytic index as the uptake rate of FITC-Zymosan particles per cell.

### *In vivo* Phagocytosis Assay

Experiments were performed using adult (10–14 weeks) wild-type C57BL/6J, PI3Kγ^−/−^, and PI3Kγ^KD/KD^ mice (seven mice per group) kept at neutral ambient temperature (*T* = 30°C) during the whole experimental period. Mice were initially injected with either low-dose LPS (0.025 mg/kg, i.p.) or high-dose LPS (10 mg/kg, i.p.) for priming and 3 days later with 10 mg/kg, i.p. LPS. Administration of FITC-labeled Zymosan particles (9,800 U/ml) was performed 24 h after the second LPS injection as described previously (26). Mice were anesthetized by intraperitoneal injection of ketamine (100 mg/kg) and xylazine (16 mg/kg) and then positioned in a stereotaxic apparatus (Stoelting, Wood Dale, IL, USA). Mice were then placed on a homoeothermic heat blanket to maintain normal body temperature during surgery. The skull was exposed by a skin incision, and small burr holes were drilled through the skull. A micromanipulator cannula (diameter, 0.24 mm) attached on a Hamilton microsyringe (10 μl) was stereotaxically placed at the parietal cortex on both sides [stereotaxic coordinates were AP, −2.0 mm; L, ±0.5 mm; and V, −2.5 mm; (27)]. Subsequently, 4 μl of FITC-labeled Zymosan particles suspended in artificial cerebrospinal fluid were infused within 120 s. The cannula remained in place for 5 min before removal. Twenty-four hours later, mice were deeply anesthetized and perfused with 4% paraformaldehyde (PFA) in phosphate buffer by cardiac puncture via the left ventricle.

Brains were removed immediately after fixation and post-fixed for 5 h in 4% PFA at 4°C. After cryoprotection in PBS containing 30% sucrose, brains were frozen in methylbutane at −30°C and stored at −80°C. Whole brains were cut by coronal sections at 40 μm on a freezing microtome (Microm International GmbH, Thermo Scientific, Germany). The slices were immunostained with anti-Iba1 antibody to visualize microglia. A voxel with an edge length of 400 μm and an altitude of 40 μm was predefined as region of interest. Z-stack imaging was performed with a 20×objective using a digital fluorescence camera (Nikon DS-Qi2), mounted on the Nikon inverted research microscope Eclipse Ti (Nikon Instruments Europe B.V., Amstelveen, The Netherlands). Quantitative measurements (ImageJ software, National Institutes of Health, Bethesda, MD) blinded to the treatment groups were used to count the percentage number of Iba-1 positive cells per cubic millimeter containing Zymosan particles.

### Blood Plasma and Brain Tissue Cytokine Measurement

Cytokine concentration in supernatants of brain tissues and blood plasma collected 24 h after the second injection LPS was measured using commercial ELISA kits obtained from BioLegend (San Diego, CA). as mentioned previously. Blood samples were collected in heparin-coated tubes (1.5 ml) by the heparinized needles and centrifuged for 10 min at room temperature at 1,500 × *g*. The plasma supernatant was immediately taken and kept at −80°C until measurement. The brain tissue was harvested after rinsing with cold PBS, immediately put in ice cold methyl butane, and kept at −80°C until processing. The brain tissue was then powdered in homogenization buffer (50 mM Tris, 150 mM NaCl, 5 mM EDTA; pH to 8.0) containing phosphatase and protease inhibitors, and then centrifuged at 10,000 × *g* for 10 min at 4°C. The supernatant was stored at −80°C until the measurement. Cytokine production data were normalized to the protein concentrations of each sample.

### Analysis of Cell Viability by MTT Assay

Cells were seeded into a 96-well plate and incubated at 37°C (5% CO_2_) for 24 h. After attachment, primary microglial cells were treated according to the stimulation scheme depicted in Figure 1A. Twenty-four hours after the second stimulation by LPS, MTT (3-(4,5-dimethylthiazol-2-yl)-2,5-diphenyltetrazolium bromide) solution was added to primed cells and incubated for 4 h at 37°C (5% CO_2_). In parallel, the same procedure was performed on US cells as well as on UP cells (stimulation occurred solely on day 6 with a fixed dose of 100 ng/ml LPS). Afterwards, the solubilization solution was added to each well and incubated overnight at 37°C (5% CO_2_) for 24 h. Absorbance was measured at 570 nm using a TECAN Infinite 200 Plate Reader (Tecan, Switzerland).

### Statistical Analysis

Data were presented as scatter dot plots, mean ± SEM. Comparisons between groups were made with one-way or two-way analysis of variance, if appropriate. *Post-hoc* comparisons were made with the Holm–Sidak test. Differences were considered significant, when *p* < 0.05. The SigmaPlot program for Windows Version 13.0 Build 13.0.0.83 (Systat Software GmbH, Erkrath, Germany) was applied for all statistical analyses.

## Results

### Priming Dose Triggers Trained Immunity and Tolerance Responses of Microglial Cells

**Figure 2:**
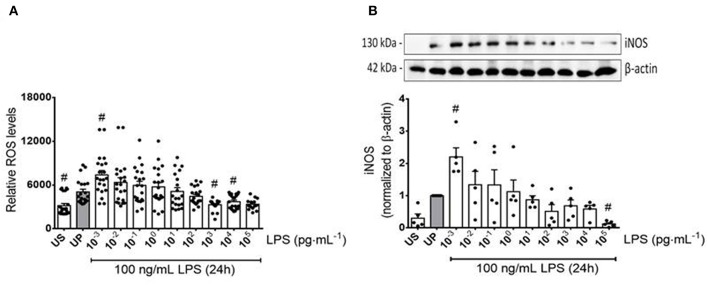
Reactive oxygen and nitrogen responses of microglia primed with increasing doses of LPS. Primary microglial cells were stimulated with increasing doses of LPS (1 fg/ml to 100 ng/ml) for 24 h and rechallenged on day 6 with a fixed dose of LPS (100 ng/ml). Supernatants and protein lysates were collected 24 h after the second stimulus by LPS. **(A)** ROS levels (*n* = 3, repeated measurements) were measured by the ROS 2′ ,7′-Dichlorodihydrofluorescein-diacetate assay. **(B)** iNOS (*n* = 5) were measured by Western blotting and quantification (unprimed cells assigned as 1.0). Data are given as scatter dot plots, mean + SEM, ^#^*p* < 0.05, ^#^significant differences vs. unprimed condition (gray column). US, unstimulated; UP, unprimed.

To verify a possible dose dependency of microglia during evolvement of trained immunity and/or immune tolerance, a widespread panel of different LPS doses for priming was used (Figures 1, 2). Primed microglial cells showed a distinct biphasic dose-response pattern for the prototypical pro-inflammatory cytokines TNF-α and IL-6, exhibiting enhanced cytokine release after low-dose priming and markedly diminished cytokine measures when microglial cells have been primed with higher LPS dosages (Figures 1B,C,E, Supplementary Figure 7). The biphasic response patterns of microglia after priming with low and high doses of LPS considerably oppose the effects of LPS dosage on the cytokine production of UP microglial cells (Supplementary Figure 1).

Both training and tolerance responses have also been found after initial priming with β-glucan and restimulation with LPS (Figure 1D). Notably, LPS doses causing training effects were considerably lower than training inducing doses of β-glucan (1 fg/ml vs. 100 fg/ml), presumably reflecting the lower threshold of LPS-induced priming in naïve neonatal microglial cells (Supplementary Figure 2). In contrast to the stimulatory effects of low doses of LPS or β-glucan on the release of the pro-inflammatory cytokines TNF-α and IL-6, the anti-inflammatory cytokine IL-10 did not exhibit any change at low doses but significantly increased after priming with high dosages of LPS.

**Figure 3:**
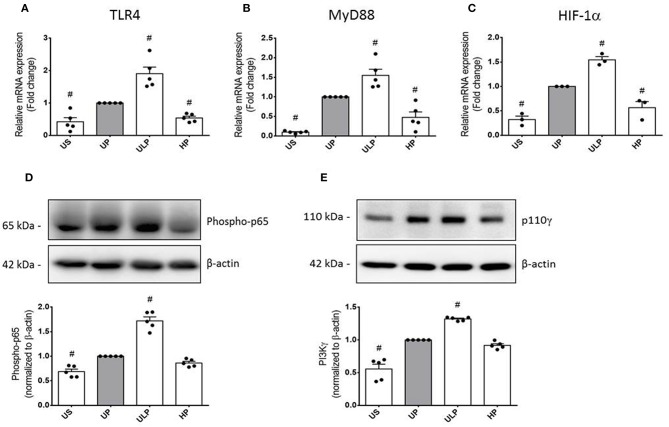
Modification of TLR4 expression and related intracellular signaling in primed microglia. Primary microglial cells were stimulated using the two-step approach with fixed priming doses of LPS [ULP (ultra-low dose, 1 fg/ml) and HP (high-dose, 100 ng/ml)]. RNA samples (6 h) and lysates (24 h) were collected after the second stimulus with LPS (100 ng/ml) and analyzed for **(A)** TLR4 expression (*n* = 5), **(B)** MyD88 expression (*n* = 5), and **(C)** HIF-1α expression (*n* = 3) by real-time PCR normalized to GAPDH representing relative values to unprimed state (assigned as 1.0). **(D)** Phospho-p65 (*n* = 5) and **(E)** PI3Kγ subunit p110γ (*n* = 5) protein expression were measured by Western blotting and quantified (unprimed cells assigned as 1.0). Data are shown as scatter dot plots, mean + SEM, ^#^*p* < 0.05, ^#^ significant differences vs. unprimed condition (gray column). US, unstimulated; UP, unprimed.

To further examine the effects of LPS dosage on the immune memory responses of primed microglia, we investigated the production of ROS and the expression of inducible NO synthase (iNOS) as the key mediator of NO production in microglia. As shown in Figures 2A,B (see also Supplementary Figure 8), both mediators of inflammatory responses revealed training and tolerance reactions at identical LPS priming doses in relation to the pro-inflammatory cytokines TNF-α and IL-6. Next, we asked for candidate mediators involved in signaling effects of increasing LPS doses used for priming of microglia. Gene expression analysis of toll-like receptor 4 (TLR4), and myeloid differentiation primary response protein 88 (MyD88) revealed again biphasic dose-dependent response with upregulation/training after low-dose priming and a significant downregulation/tolerance effect after priming with high-dose LPS (Figures 3A,B). These findings were confirmed by quantification of protein expression (Supplementary Figures 3, 13, 14). PI3Kγ mutations were without relevant effects (Supplementary Figure 4). Similar priming effects of increasing doses of LPS have been observed for the hypoxia-inducible factor-1a (HIF-1α) gene expression (Figure 3C). A concordant activity change of the NFκB subunit p65 was observed, verified by corresponding phosphorylation states (Figure 3D, Supplementary Figure 9). Finally, low-dose LPS priming of microglial cells was shown to provoke an upregulation of the inflammatory mediator PI3Kγ compared to UP cells (Figure 3E, Supplementary Figure 10).

### Role of PI3Kγ in Immune Memory Responses of Microglia

The involvement of PI3Kγ in trained immunity and immune tolerance provoked by increasing LPS dosages has been further elaborated by comparative analysis of microglia isolated from wild-type mice, mice deficient in PI3Kγ expression (PI3Kγ^−/−^), and mice expressing a catalytically inactive mutant of PI3Kγ (PI3Kγ^KD/KD^).

**Figure 4:**
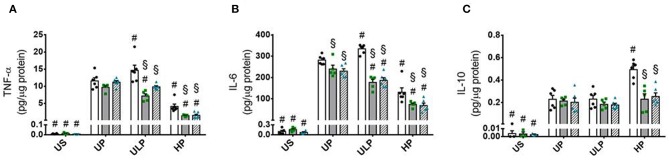
PI3Kγ mediates innate immune memory cytokine response of microglia. Primary microglial cells (wild-type •, open columns; PI3Kγ^−/−^ ■, dark gray columns; PI3Kγ^KD/KD^ ▴, hatched columns) were primed with fixed priming doses of LPS [ULP (ultra-low dose, 1 fg/ml) and HP (high-dose, 100 ng/ml)] and restimulated on day 6 with a fixed dose LPS (100 ng/ml). Supernatants were collected 24 h after the second stimulation by LPS and the cytokine levels for **(A)** TNF-α, **(B)** IL-6, and **(C)** IL-10 were assayed by ELISA (normalized to total protein concentration). Data are shown as scatter dot plots, mean + SEM, *n* = 6, ^#^*p* < 0.05, ^#^significant differences vs. unprimed condition (UP) and ^§^*p* < 0.05, ^§^significant differences vs. microglia derived from wild-type mice. US, unstimulated.

**Figure 5:**
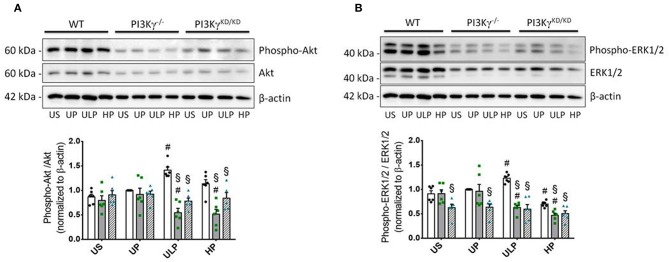
PI3Kγ mediates Akt and Erk signaling in primed microglia. Primary microglial cells (wild-type •, open columns; PI3Kγ^−/−^ ■, dark gray columns; PI3Kγ^KD/KD^ ▴, hatched columns) were primed with fixed priming doses of LPS [ULP (ultra-low dose, 1 fg/ml) and HP (high-dose, 100 ng/ml)] and restimulated on day 6 with a fixed dose LPS (100 ng/ml). Lysates were collected 24 h after the second stimulation by LPS and the protein expression of **(A)** phospho-Akt and **(B)** phospho-ERK1/2 were assayed by Western blotting and quantified (unprimed cells assigned as 1.0). Data were presented as scatter dot plots, mean + SEM, *n* = 6, ^#^*p* < 0.05, ^#^significant differences vs. unprimed condition (UP) and ^§^*p* < 0.05, ^§^significant differences vs. microglia derived from wild-type mice. US, unstimulated.

Both genotypes of mice allow assignment of observed effects of increasing LPS doses on inflammatory responses of microglia to either lipid kinase function of PI3Kγ (23) or kinase-independent “scaffold” functions, such as PDE stimulation and cellular cAMP control (20, 28). The observed patterns of cytokine release revealed that the lipid kinase function of PI3Kγ appears to be responsible for the dose-dependent priming effects of LPS on microglial cells. Both PI3Kγ^−/−^ and PI3Kγ^KD/KD^ microglia revealed downregulation of pro-inflammatory cytokines after low-dose priming and a further reduction of the inflammatory response after high-dose priming (Figures 4A,B). Furthermore, increased production of IL-10 after high-dose LPS priming in wild-type microglia revealed dependency on enzymatic activity of PI3Kγ (Figure 4C). In line with these observations, activation of protein kinase B/Akt as the key downstream mediator of PI3Kγ lipid kinase activity disclosed the already described dose-dependent biphasic priming effects of LPS on microglial cells with enhanced Akt phosphorylation after low-dose priming. Both PI3Kγ mutants exhibited a suppressed Akt phosphorylation state (Figure 5A, Supplementary Figure 11). In addition, the biphasic effects of dose-dependent priming by LPS in wild-type microglial cells have also been observed for the mitogen-activated protein kinase (MAPK)-mediated pro-inflammatory pathway as shown by the phosphorylation states of the extracellular signal–regulated kinase (ERK) 1/2 (Figure 5B, Supplementary Figure 12). Consistently, both PI3Kγ mutant microglia exhibited a suppressed ERK1/2 phosphorylation state.

In mice, LPS priming exhibited dose-dependently systemic and cerebral inflammatory responses. Measurement of the pro-inflammatory cytokines TNF-α and IL-6 in blood revealed a remittent systemic inflammation response syndrome (SIRS) (Table 1) indicated by rather low cytokine concentrations with a distinct reduction in both PI3Kγ mutants for all cytokines under consideration. Furthermore, high-dose priming showed a tolerance effect for TNF-α in wild-type mice, whereas IL-10 was reduced after low-dose priming and increased after high-dose priming compared with single LPS administration. In contrast, SIRS induced a maintained inflammatory response in brain tissue with obvious priming effects: Whereas single LPS administration induced similar effects of pro- and anti-inflammatory cytokine concentrations under consideration in wild-type and both PI3Kγ mutant mice, wild-type mice exhibited a distinct elevation of pro-inflammatory cytokines TNF-α and IL-6 due to low-dose priming and increased IL-10 after high-dose priming, but these effects were missed in both PI3Kγ mutants (Table 1).

**Table 1:** Cytokine content in blood plasma and brain tissue.

	**Cytokines**		**Unprimed**	**Low-dose priming**	**High-dose priming**
**Blood plasma**					
	TNF-α (ng·g^−1^)	WT	7.8 (6.6; 9.2)	7.0 (6.9; 9.6)	4.3 (2.6; 4.4)^*^
		PI3Kγ^−/−^	2.6 (1.7; 3.0)^§^	1.3 (0.5; 4.2)^§^	1.7 (1.4; 2.0)^§^
		PI3Kγ^KD/KD^	1.7 (0.6; 2.4)^§^	0.8 (0.5; 1.0)^§^	0.8 (0.5; 1.2)^§^
	IL-6 (ng·g^−1^)	WT	19.4 (17.1; 20.2)	23.6 (21.7; 25.9)^*^	16.1 (13.3; 19.3)
		PI3Kγ^−/−^	9.5 (7.9; 12.1)^§^	4.9 (3.8; 7.9)^§^	8.4 (6.8; 10.9)^§^
		PI3Kγ^KD/KD^	11.6 (5.9; 13.3)^§^	5.5 (4.8; 7.3)^§^	3.3 (2.9; 7.5)^§^
	IL-10 (ng·g^−1^)	WT	7.0 (5.2; 9.0)	2.0 (1.8; 2.1)^*^	18.6 (17.8; 18.7)^*^
		PI3Kγ^−/−^	0.4 (0.0; 1.2)^§^	1.0 (0.5; 1.2)	0.0 (0.0; 0.8)^§^
		PI3Kγ^KD/KD^	0.0 (0.0; 0.7)^§^	n.d.	0.6 (0.0; 1.5)^§^
**Brain tissue**					
	TNF-α (ng·g^−1^)	WT	30.6 (26.2; 37.6)	59.2 (54.3; 67.6)^*^	31.3 (23.5; 34.1)
		PI3Kγ^−/−^	29.8 (20.1; 35.1)	25.5 (24.4; 27.0)^§^	36.5 (35.8; 40.9)
		PI3Kγ^KD/KD^	28.4 (25.3; 30.0)	26.0 (25.0; 29.7)^§^	28.6 (26.8; 30.0)
	IL-6 (ng·g^−1^)	WT	99.0 (79.8; 109.1)	122.7 (119.1; 128.3)^*^	74.9 (61.0; 78.3)^*^
		PI3Kγ^−/−^	78.4 (75.6; 86.0)	73.5 (68.0; 75.7)^§^	74.2 (65.6; 83.5)
		PI3Kγ^KD/KD^	89.7 (57.7; 95.7)	78.0 (71.9; 83.8)^§^	60.9 (55.3; 68.8)
	IL-10 (ng·g^−1^)	WT	214 (212; 218)	220 (209; 234)	318 (311; 325)^*^
		PI3Kγ^−/−^	176 (174; 229)	174 (158; 187)^§^	174 (173; 178)^§^
		PI3Kγ^KD/KD^	183 (133; 240)	172 (156; 183)	165 (162; 178)^§^

Values are given as medians as well as the first quartile and third quartile in parenthesis, p < 0.05, ^*^significant differences vs. “Unprimed” within the same genotype, ^§^significant differences vs. wild-type (WT) mice within the same condition.

**Figure 6:**
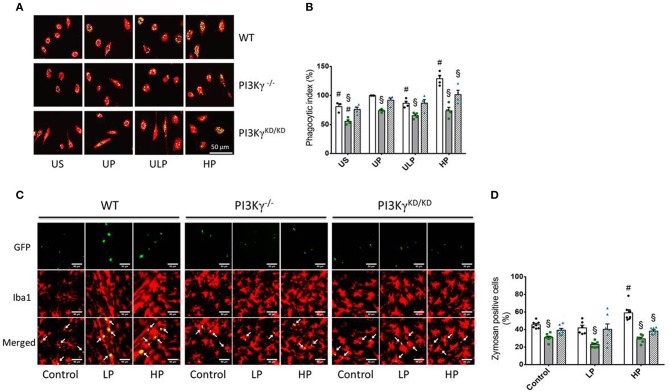
PI3Kg mediates phagocytic activity of microglial cells after priming with LPS in cell culture and mouse brain. **(A,B)** Primary microglial cells [wild-type (WT), •, open columns; PI3Kγ^−/−^ ■, dark gray columns; PI3Kγ^KD/KD^ ▴, hatched columns] were stimulated using the two-step approach with fixed priming doses of LPS [ULP (ultra-low dose, 1 fg/ml) and HP (high-dose, 100 ng/ml)] and restimulated on day 6 with a fixed dose LPS (100 ng/ml). Twenty-four hours later, cells were incubated with fluorescein isothiocyanate (FITC)-labeled Zymosan A for 1 h. Iba1 was used as a marker of primary microglial cells (red color). DAPI (blue) was used for staining the nucleus of the cells and GFP for labeling FITC-Zymosan particles. Quantification of *in vitro* phagocytosis **(B)** (*n* = 4) was performed as uptake rate of FITC-Zymosan particles per cell (phagocytic index). **(C)** Mice [wild-type (WT), •, open columns; PI3Kγ^−/−^ ■, dark gray columns; PI3Kγ^KD/KD^ ▴, hatched columns] were initially injected with either low-dose LPS (LP, 0.025 mg/kg, i.p.) or high-dose LPS (HP, 10 mg/kg, i.p.) for priming and 3 days later with a subsequent application of 10 mg/kg, i.p. LPS. Administration of FITC-labeled Zymosan particles (9,800 U/ml) was performed 24 h after the second LPS injection. Brains were harvested and processed 24 h later. Iba1 (microglial cells) and GFP (FITC-labeled Zymosan particles) were used as markers. Quantification of phagocytosis **(D)** was performed as percentage of Iba-1 positive cells per voxel mm^3^ containing Zymosan particles (*n* = 7). Data are shown as scatter dot plots, mean + SEM, ^#^*p* < 0.05, ^#^significant differences vs. unprimed condition (*in vitro*) or vs. controls (single injection with 10 mg/kg, i.p. LPS; *in vivo*); ^§^significant differences vs. microglia derived from wild-type mice or WT mice of the same treating group. US, unstimulated; UP, unprimed; LP, low primed.

To test whether priming induced immune memory influences microglial phagocytosis as a key macrophage function, we assessed microglial phagocytic activity in cell culture and *in vivo* experiments. The *in vitro* studies revealed that LPS treatment resulted in a significant increase in phagocytic activity in UP wild-type microglia (Figure 6B). UP microglia exhibit an inhibition of phagocytosis in PI3Kγ^−/−^ microglia as reported previously (15). Similar effects emerge after low-dose priming and subsequent LPS stimulation (Figure 6). Notably, high-dose priming induced an increased phagocytic activity in microglia derived from wild-type mice as well as when zymosan particles were administrated into the brain of wild-type mice. In contrast, both PI3Kγ mutant mice showed decreased phagocytosis of these particles at high LPS priming dose, indicating a key regulatory function of PI3Kγ lipid kinase activity under these conditions (Figures 6C,D).

## Discussion

The discovery of memory-like activities of the innate immune system attracts increasing interest in immunology, infection biology, and medicine. Both trained immunity and immune tolerance have been described as enduring response patterns of innate immune cells to PAMPs (29, 30). Initial studies of tolerance and training phenomena mostly addressed adaptive responses of peripheral immune cells. Intriguingly, it has been shown that innate monocytes and macrophages can be dynamically programmed into distinct states depending on the strength and duration of classical pattern recognition molecules like LPS (31). Increased expression of pro-inflammatory mediators was observed in monocytes primed with lower doses of LPS and suppressed expression in monocytes primed with higher doses of LPS (32, 33). Recent work also revealed immune memory for microglia, the brain-resident macrophages (17, 18). Treating primary microglia with an initial LPS challenge of 100 ng/ml for 24 h, Schaafsma et al. found a blunted pro-inflammatory response, i.e., tolerance development as the consequence of a subsequent LPS stimulation after a 6-days interval. Wendeln et al. used daily peritoneal treatment of mice with LPS (daily dose of 500 μg/kg bodyweight) for monitoring the cytokine response in the brain and observed effects of trained immunity a few hours after the second day of LPS challenge. In contrast, immune tolerance occurred after 4 days of LPS administration. These seminal contributions identified epigenetic modifications as causal contributors for long-term innate memory effects in the brain and as important modifiers of neuropathology. Despite indications of expression changes of genes regulating epigenetic modifications after LPS priming and subsequent restimulation (Supplementary Figure 6), from our point of view, both studies provoke two intriguing questions: (i) Are microglial cells indeed unable to express features of trained immunity under *in vitro* conditions? (ii) What are the causes for consecutive training and tolerance development shown in mice brain microglia?

Our data move the dosage of PAMPs and pathogens into the focus. As shown in Figure 1, extremely low doses of LPS express ability to induce sensitization, i.e., trained immunity of isolated microglial cells 1 week after the initial treatment. The training effect not only is restricted to classical pro-inflammatory cytokines like TNF-α and IL-6 but also involves key mediators of the inflammatory response like ROS, the expression of iNOS, and the expression of the PAMP receptor TLR-4 and its associated protein MyD88. Furthermore, p65 phosphorylation as a key element of the NFκB signaling pathway was shown to exhibit pronounced training response after microglial priming with femtomolar LPS concentrations. Sensitization of NFκB by priming with a lower LPS dosage and tolerance development by a higher LPS dose has been recently observed by single-cell analysis and assigned to a dose-sensing autoregulatory loop via IL-1R-associated kinase 1 (IRAK1) (34). Finally, under these conditions, increased expression of HIF-1α and PI3Kγ was induced; both of them are central mediators of inflammatory responses (19, 35, 36). Together, these data indicate robust pro-inflammatory overactivation by priming with ultra-low LPS dosage as a key characteristic of trained immunity in microglia.

Training responses of microglia primed with ultra-low LPS dose may raise the question of the physiological relevance of these experimental conditions. Previous studies revealed extraordinary potency of endotoxins like LPS mediating host responses of 100 invading Gram-negative bacteria, which corresponds to femtomoles of endotoxin (1, 2, 37). LPS is known to be a well-characterized PAMP found in the outer leaflet of the outer membrane of most of the Gram-negative bacteria. The structurally unique lipid A region of LPS is the principal determinant of this pro-inflammatory activity. Sensitive LPS-induced immune cell activation is normally mediated by plasma membrane bound receptor TLR4 (38, 39). Potent activation of TLR4 by LPS requires initial interactions of LPS-binding protein and CD14 with LPS-rich interfaces followed by extraction and transfer of individual LPS monomers first to CD14 (3, 40, 41). Activation of MD-2/TLR4 by LPS requires binding of an individual LPS molecule (LPS monomer) to MD-2/TLR4 (40) and dimerization of the LPS.MD-2.TLR4 ternary complex (42). Recent sophisticated studies disclosed that as few as 25 LPS molecules provoke oligomerization of TLR4 complexes per cell, which can trigger measurable pro-inflammatory responses (43). Consequently, priming effects of ultra-low PAMP doses might be sufficiently effective to induce innate immune memory responses.

Interestingly, priming with ultra-low LPS dose did not induce accompanying anti-inflammatory response as indicated by a missing IL-10 elevation (Figures 1F, 4C). The reason for these findings cannot be drawn from our results. Nevertheless, it can be speculated that neonatal microglial cells respond to priming with ultra-low LPS doses like leukocytes with non-resolving inflammatory adaptation (33). Yuan and coworkers disclosed that the TRAM/TRIF pathway represents the key circuit involved in the low-grade inflammatory adaptation of monocytes and may be responsible for the removal of negative modulators, such as the transcriptional modulator B lymphocyte-induced maturation protein-1 (Blimp-1), thus disallowing the development of anti-inflammatory tolerance and favoring the inflammatory monocyte adaptation.

Immune tolerance in brain has been shown to be involved in brain tissue responses on acute injuries like ischemia or trauma induced by a panel of noxious stimuli including neuroinflammation near but below the threshold of damage (36, 44–46). Modified TLR-4 signaling toward activation of the interferon regulatory factor IRF3 and activation of microglial cells are clearly key elements in tolerance like preconditioning effects (46–48). In our hands, immune tolerance of microglia arises subsequent to repeated treatment of microglia cells with high doses of LPS characterized by decreased expression of pro-inflammatory cytokines and accompanied by increased level of anti-inflammatory IL-10 in comparison to UP controls. These observations confirm previous findings (17) and fit to an energy-saving phenotype of microglia at high pathogen load. Exposed to high PAMPs doses, these innate immune cells evidently turn down energy-demanding anabolic resistance reactions and switch to tolerance responses accompanied by less energy-demanding catabolic processes (29, 49). Nevertheless, the high dose tolerance effects (e.g., reduced expression and release of pro-inflammatory cytokines, elevated phagocytic activity) might reflect mainly a regulatory response resulting from IL-10 upregulation. This consideration derived from our data is in line with previous reports and may preclude an exhaustion of the machinery to produce pro-inflammatory cytokines [for review, see (50)]. In contrast to primed microglia, we found in UP microglia a dose-dependent increase of pro-inflammatory cytokines indicating increased ability to produce pro-inflammatory cytokines together with anti-inflammatory IL-10 (Supplementary Figure 1). Moreover, the increased phagocytic activity after high-dose LPS priming compared to the UP state indicates a regulatory effect and rather precludes an exhaustion of the system (Figure 6). Furthermore, results of the MTT assay (Supplementary Figure 5) verified maintenance of cellular integrity even after high-dose LPS priming. Our findings are in line with previous reports. Jack et al. (51) showed that human microglia exposed to LPS become refractory to a secondary stimulation with the ligand, indicative of a strict negative regulation (51). Park et al. (52) reported that LPS-induced endogenous expression of IL-10 in microglia contributes to neuronal survival by inhibiting brain inflammation after initial pro-inflammatory activation (52).

We have to recognize that it is well-known that microglial cells contribute to the onset and progression of neuroinflammation, but it is only now becoming apparent that these resident immunocompetent cells also play an important role in producing and responding to immunosuppressive factors that serve to limit the detrimental effects of an overwhelming neuroinflammation (53). The herein documented induction of immune tolerance induced by repeated high-dose LPS stimulation mimics consequences of repeated neuroinflammation and suggests that robust primary CNS affection by PAMPs led to a memory effect for decreased response to a secondary PAMP confrontation. Therefore, immune tolerance represents an altered microglial phenotype characterized by an altered response dynamics. Indeed, the time frame of a single PAMP-induced neuroinflammatory response is composed of an initial massive pro-inflammatory response peaking within few hours followed by a resolution phase induced by delayed secondary production of mediators that are immunosuppressive and/or neuroprotective 24–48 h later (51, 54). Jack and coworkers also revealed evidence for a feedback loop involving significant downregulation of the TLR-4 expression, but not TLR-2. Importantly, these authors primed human fetal microglia with 10 ng/ml LPS for 24 h followed by LPS 100 ng/ml challenge and observed significantly low levels of TNFα and IL-6 in primed microglia in comparison with non-primed microglia. This could be the first evidence of tolerance development induced by low-dose priming with LPS (51).

Together, our results identify the concentration of LPS as the critical mediator of trained immunity and immune tolerance in microglia. The herein reported data suggest induction of a general long-term reaction pattern that predominantly depends on the dose of PAMPs sensed by microglial cells. Dose-dependent development of microglial trained immunity and immune tolerance might appear as a “hormetic” principle underlying the pathogen-induced response of the innate immune system (29, 55, 56).

As a rationale of the pathogen-dose-dependent responses of training and immune tolerance, differential energy investment might contribute to the observed reaction patterns. Innate immune cells challenged with low amounts of bacterial pathogens can generate energy-demanding resistance responses aiming at pathogen elimination. Under these conditions, cellular resources remain adequate to maintain production of cytokines, antimicrobial peptides, and other defense mediators. By contrast, higher pathogen loads might increasingly impair affected immune cells by overstretching their inherent capacity to withstand the cytotoxic effects of the stressful stimulus (29, 57). To facilitate survival, immune cells consequently shift from resistance reactions toward maintenance and repair activities, which ultimately enable tolerance toward pathogens. Hence, gradual induction of trained resistance and tolerance by increasing pathogen concentrations achieve basic fitness and survival requirements of cells and organs challenged by various PAMPs and other stressors. The dose-dependent response of microglia is not restricted to the paradigmatic PAMP LPS. Our data reveal that the fungal cell wall constituent β-glucan also provokes dose-dependent training and tolerance responses. Notably, a remarkable shift of the sensitivity of the affected microglial cells becomes apparent. Whereas, LPS-induced training was observed at 1 fg/ml LPS, β-glucan provokes training at 100 fg/ml. Different effective concentrations of LPS and β-glucan might be reasoned by varying affinities of the TLR-4 and Dectin-1 receptors binding these two PAMPs. The high sensitivity of microglia to LPS might be a special characteristic of neonatal cells (58).

Increased expression of PI3Kγ induced by low-dose priming of naïve microglia indicates involvement of the signaling protein in training effect. Indeed, PI3Kγ was identified as the major PI3K catalytic isoform in primary myeloid cells, as these cells express at least 25-fold more PI3Kγ than other isoforms (59). Furthermore, PI3Kγ has been previously shown as mediator of microglial motility, phagocytosis, and matrix metalloproteinase release using PIP3-related as well as cAMP-driven signaling pathways (15, 60, 61). Our data suggest PI3Kγ/AKT signaling as mediator for increased production of inflammatory cytokines TNF-α and IL-6 in trained microglial cells. Intriguingly, increased release of the anti-inflammatory cytokine IL-10 induced after high-dose priming was likewise dependent on PI3Kγ enzymatic activity. A similar response pattern as for Akt phosphorylation has been observed for phospho-ERK1/2. In wild-type microglial cells, low-dose LPS priming provokes stimulation of ERK phosphorylation, whereas inhibition of these signals has been observed after high-dose LPS priming. Both at low- and high-dose LPS, the ERK phosphorylation was diminished in cells deficient of PI3Kγ lipid kinase activity.

A contribution of PI3K signaling proteins in mediating inflammatory responses as a result of super-low-dose LPS has also been verified previously for human monocytes and murine bone marrow-derived macrophages (62–64). These data clearly revealed that the mild but significant pro-inflammatory response of leukocytes induced by super-low-dose LPS is apparently mediated by an insufficient induction of negative regulators of NF-κB and the PI3K/Akt pathways. Our findings indicate that ultra-low LPS priming and subsequent high-level LPS stimulation for 24 h induce an enhanced PI3Kγ lipid kinase activity in neonatal microglia with reinforced p65 phosphorylation indicative of selective upregulation of NF-κB transcriptional activity (65, 66) possibly mediated by AKT-induced IKKα phosphorylation (67).

Together, our results propose PI3Kγ as an essential mediator of cytokine responses of microglia dependent on the specific immune context (68). Both pro- and anti-inflammatory signaling functions of PI3Kγ become apparent, supporting recent reports on ambivalent regulatory functions of PI3Kγ in inflammatory processes (69, 70).

Phagocytosis of pathogens represents the eponymous function of microglia and other cells of the macrophage family. The engulfment of extracellular microbes or related particles, i.e., xenophagy (71), is closely related to autophagy and maintenance and repair processes, which are part of the tolerance responses of innate immune cells (72–74). Intriguingly, LPS treatment resulted in a significant increase in phagocytic activity in UP wild-type microglia (see Figure 5A), confirming recent results on enhanced phagocytosis induced by high-dose LPS priming (17).

Increased phagocytosis goes parallel with decreased expression of pro-inflammatory cytokines and corresponding immune tolerance. Notably, increased phagocytosis induced by high doses of LPS strictly depends on PI3Kγ. PI3Kγ-deficient cells or microglia expressing the catalytically inactive version of PI3Kγ do not exhibit increased phagocytic activity assayed *in vitro* and *in vivo*. These investigations are in line with our previous data on an essential role of PI3Kγ in microglia phagocytosis (15, 75).

## Conclusion

Collectively, our data suggest a key role of the pathogen dose in memory-like adaptive responses of microglial cells. Sequential development of trained immunity and immune tolerance induced by increasing doses of pathogen might have important implications for infection biology and medicine. Trained immunity effects at low pathogen doses might facilitate fast and efficient resistance responses toward repeated attacks of pathogens, especially in individuals lacking adaptive immunity, such as infants or immunosuppressed patients. By contrast, tolerance responses connected with repair and maintenance reactions might represent the adequate reply to damaging effects of high pathogen doses preventing a potential exhaustion of (immune) cell energy resources by escalating resistance responses.

Significant translational potential resides in research activities directed toward better understanding both components of innate immune memory. Identification of the pathogen dosage and molecular mechanisms that shape innate immune responses into specific stress response patterns is likely to be crucial for these emerging investigations.

## Data Availability Statement

The datasets generated for this study can be found in the NCBI's Gene Expression Omnibus, GEO Series accession number GSE137741.

## Ethics Statement

The animal study was reviewed and approved by The Thuringian State Office for Food Safety and Consumer Protection.

## Author Contributions

TL, RW, and RB designed the research and drafted the manuscript. DW provided the *Candida albicans*-derived β-glucan. TL, G-PL, and FH acquired the experimental data. TL and RB performed the statistical analysis. MG performed and analyzed the RNA sequencing. All authors critically revised the manuscript.

### Conflict of Interest

The authors declare that the research was conducted in the absence of any commercial or financial relationships that could be construed as a potential conflict of interest.
