# Supplementary material for: Memory-Like Inflammatory Responses of Microglia to Rising Doses of LPS: Key Role of PI3Kγ
Source: Front Immunol. 2019 Nov 8;10:2492. doi: 10.3389/fimmu.2019.02492 (PMC6856213; doi:10.3389/fimmu.2019.02492)
Supplement: Supplementary file 1 [file Data_Sheet_1.docx]

Lajqi et al. Memory-like inflammatory responses of microglia to rising doses of LPS: Key role of PI3Kγ

**Methods**

**RNA sequencing and data analysis**

Total RNA was extracted using QIAzol Lysis Reagent (#79306) purchased from Qiagen (Hilden, Germany). RNA concentration and quality were checked by using the Nanodrop ND-1000 machine (Peqlab, Erlangen, Germany).

Sequencing of RNA samples was done using Illumina’s next-generation sequencing methodology (Bentley et al., 2008). In detail, quality check and quantification of total RNA was done using the Agilent Bioanalyzer 2100 in combination with the RNA 6000 nano kit (Agilent Technologies). For library preparation 1µg of total RNA was introduced to the TruSeq Stranded mRNA Kit (Illumina) following the manufacturer’s description. Quantification and quality check of libraries was done using the Agilent Bioanalyzer 2100 in combination with the DNA 7500 kit. Libraries were sequenced on a HiSeq2500 running in 51cycle/single-end/high-output mode. Libraries were pooled and sequenced on two lanes. Sequence information was extracted in FastQ format using Illumina’s bcl2FastQ v1.8.4. Sequencing resulted in around 31mio reads per sample.

Reads were mapped to the Ensembl (Zerbino et al., 2018) mouse reference genome GRCm39.85 using TopHat2.1.0 (Kim et al., 2013). The reads mapped to the reference were counted per gene using FeatureCounts v1.5.0 (Liao et al., 2014). Resulting counts were introduced in the statistical environment R (Team, 2019) in order to calculate RPMs (read per million mappable reads) and RPKMs (reads per million mappable reads and kilobase gene length). RPKMs of selected genes were plotted in a boxplot for UP, ULP, and HP samples using R packages “ggplot2” and “cowplot”.

**Data availability**

The RNA sequencing data discussed in this publication have been deposited in NCBI's Gene Expression Omnibus and are accessible through GEO Series accession number GSE137741.


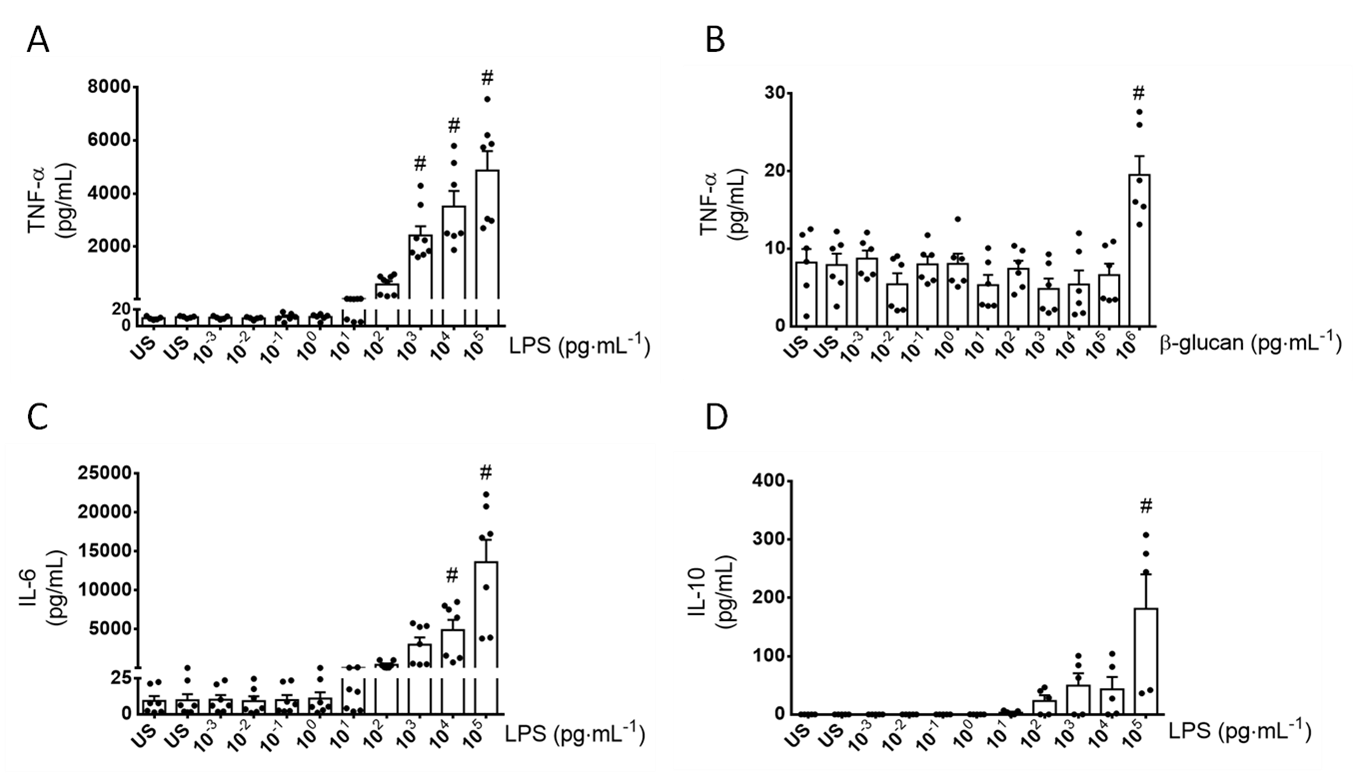


Supplementary Figure 1: **LPS and β-glucan induce dose-dependent cytokine production in unprimed microglial cells.**

Primary microglial cells were stimulated with increasing doses of LPS (1 fg/mL – 100 ng/mL) or β-glucan (1 fg/mL – 1 μg/mL) for 24 hours. Cytokine levels for (**A, B**) TNF-α (n=6-8), (**C**) IL-6 (n=7), and (**D**) IL-10 (n=5) have been assayed by ELISA. Data are shown as scatter dot plots, mean + SEM, #p <0.05, # vs. unstimulated (US) condition.


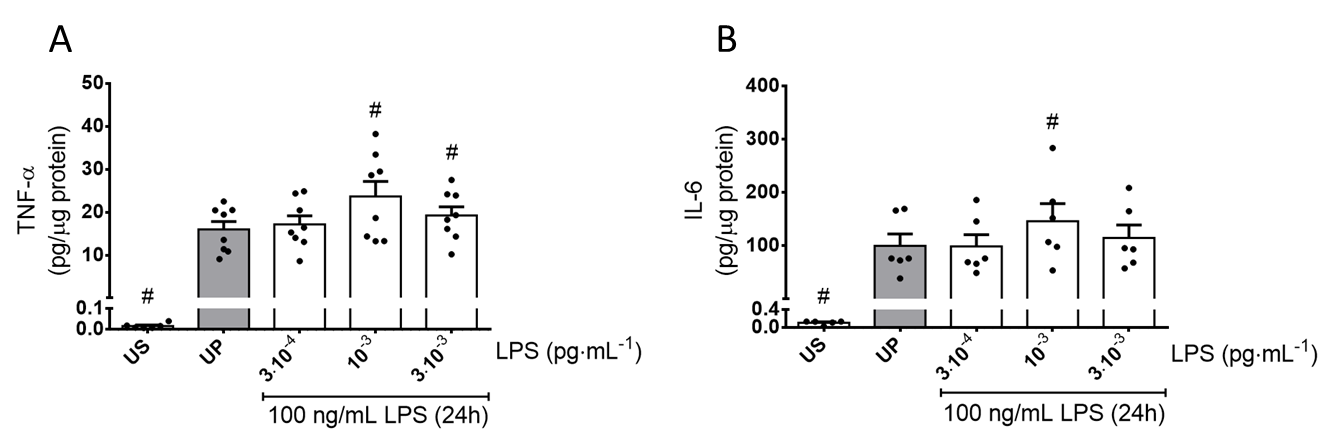


Supplementary Figure 2: **Estimation of the lower threshold of LPS-induced priming on cytokine responses in naïve neonatal microglial cells.**

Primary microglial cells were stimulated using the two-step approach with fixed priming doses of LPS with 0.3 fg/mL, 1 fg/mL and 3 fg/mL and re-stimulated on day 6 with a fixed dose LPS (100 ng/mL). Supernatants were collected 24h after the 2nd stimulation by LPS and cytokine levels (A: TNF-α, n=8; B: IL-6, n=6) were assayed by ELISA (normalized to total protein concentration). Data were presented as scatter dot plots, mean + SEM, #p <0.05, # significant differences versus unprimed condition (UP) wild-type mice. US, unstimulated.


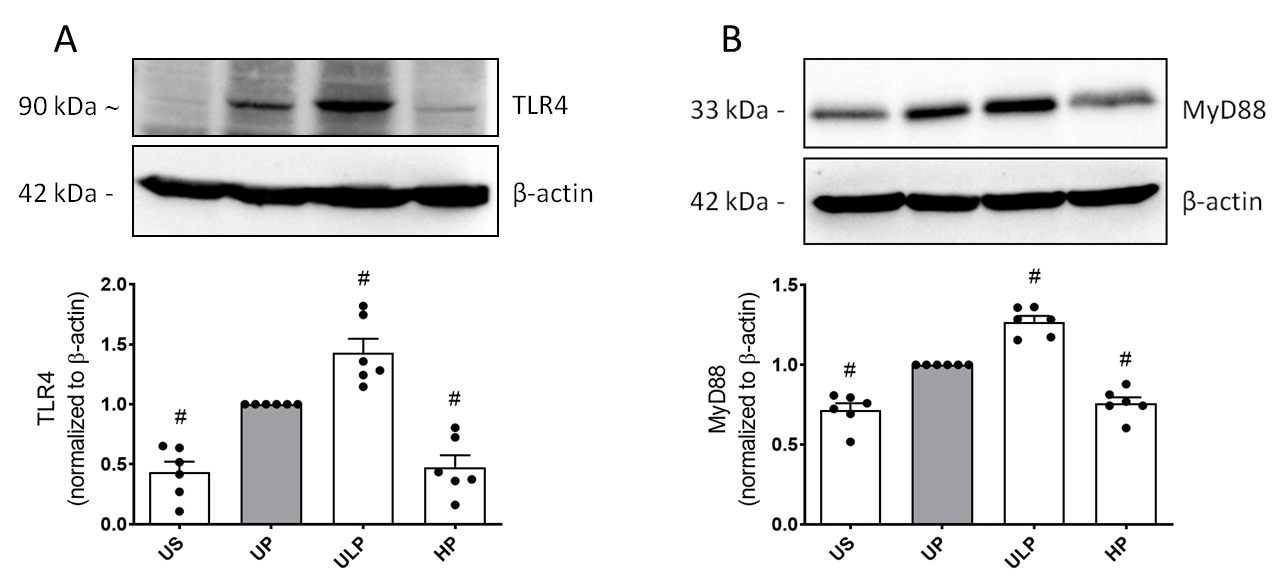


Supplementary Figure 3: **Modification of TLR4 and MyD88 protein production in primed microglia.**

Primary microglial cells were stimulated using the two-step approach with fixed priming doses of LPS [ULP (ultra-low dose, 1 fg/mL) and HP (high-dose, 100 ng/mL)] and re-stimulated on day 6 with a fixed dose LPS (100 ng/mL). Lysates were collected 24h after the 2nd stimulation by LPS and the protein expression of (A) TLR4 and (B) MyD88 were assayed by Western blotting and quantified (unprimed cells assigned as 1.0). Data were presented as scatter dot plots, mean + SEM, n=6, #p <0.05, # significant differences versus unprimed condition (UP). US, unstimulated.


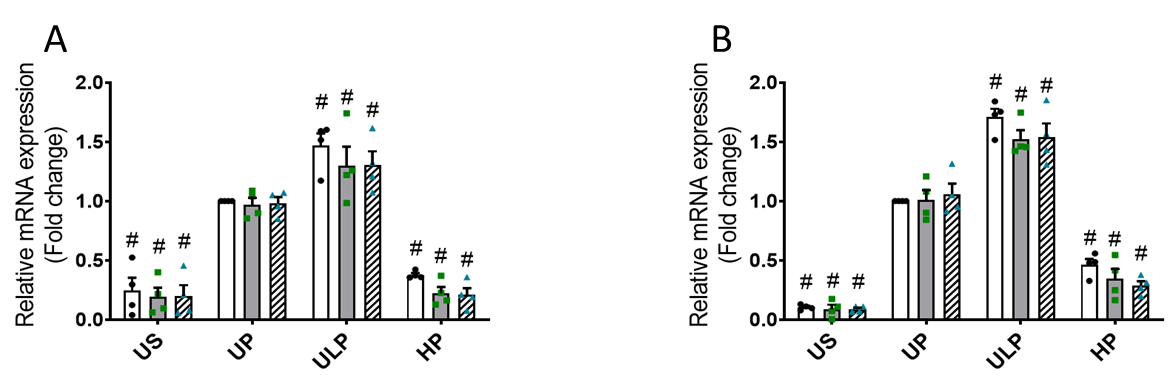


Supplementary Figure 4: **Modification of TLR4 and MyD88 expression in primed microglia derived from wild type and PI3Kγ mutant mice.**

Primary microglial cells (wild-type ●, open columns; PI3Kγ^-/-^ ■, dark gray columns; PI3Kγ^KD/KD^ ▲, hatched columns) were stimulated using the two-step approach with fixed priming doses of LPS [ULP (ultra-low dose, 1 fg/mL) and HP (high-dose, 100 ng/mL)] and re-stimulated on day 6 with a fixed dose LPS (100 ng/mL). RNA samples were collected 6h after the 2nd stimulation by LPS and the protein expression of (A) TLR4 and (B) MyD88 were assayed by real-time PCR normalized to GAPDH representing relative values to unprimed state. Data are shown as scatter dot plots, mean + SEM, n=4, ^#^p <0.05, ^#^ significant differences versus unprimed condition. US, unstimulated; UP, unprimed.


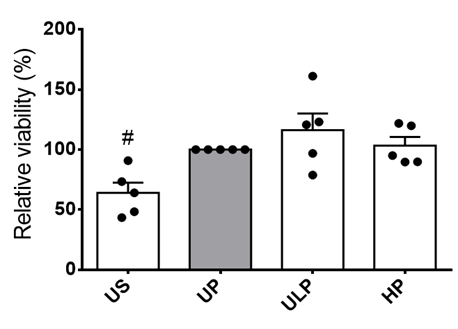


Supplementary Figure 5: **Analysis of cell viability in primed microglia by the MTT assay.**

Primary microglial cells were stimulated using the two-step approach with fixed priming doses of LPS [ULP (ultra-low dose, 1 fg/mL) and HP (high-dose, 100 ng/mL)] and re-stimulated on day 6 with a fixed dose LPS (100 ng/mL). MTT (3-(4,5-dimethylthiazol-2-yl)-2,5-diphenyltetrazolium bromide) solution was added to primed cells and incubated for 4 h at 37 °C (5 % CO_2_). In parallel, same procedure was performed on unstimulated (US) cell as well as on unprimed (UP) cells (stimulation occurred solely on day 6 with a fixed dose of 100 ng/mL LPS). Afterwards the solubilization solution were added to each well and incubate overnight at 37 °C (5 % CO2) for 24 h and absorbance was measured at 570 nm. Data were normalized to the unprimed state. Data are shown as scatter dot plots, mean + SEM, n=5, ^#^p <0.05, ^#^ significant differences versus unprimed condition (grey column). US, unstimulated; UP, unprimed.

Supplementary Figure 6: **Expression** **analysis of genes involved in epigenetic modifications in murine microglial BV-2 cells after LPS priming and subsequent re-stimulation**.


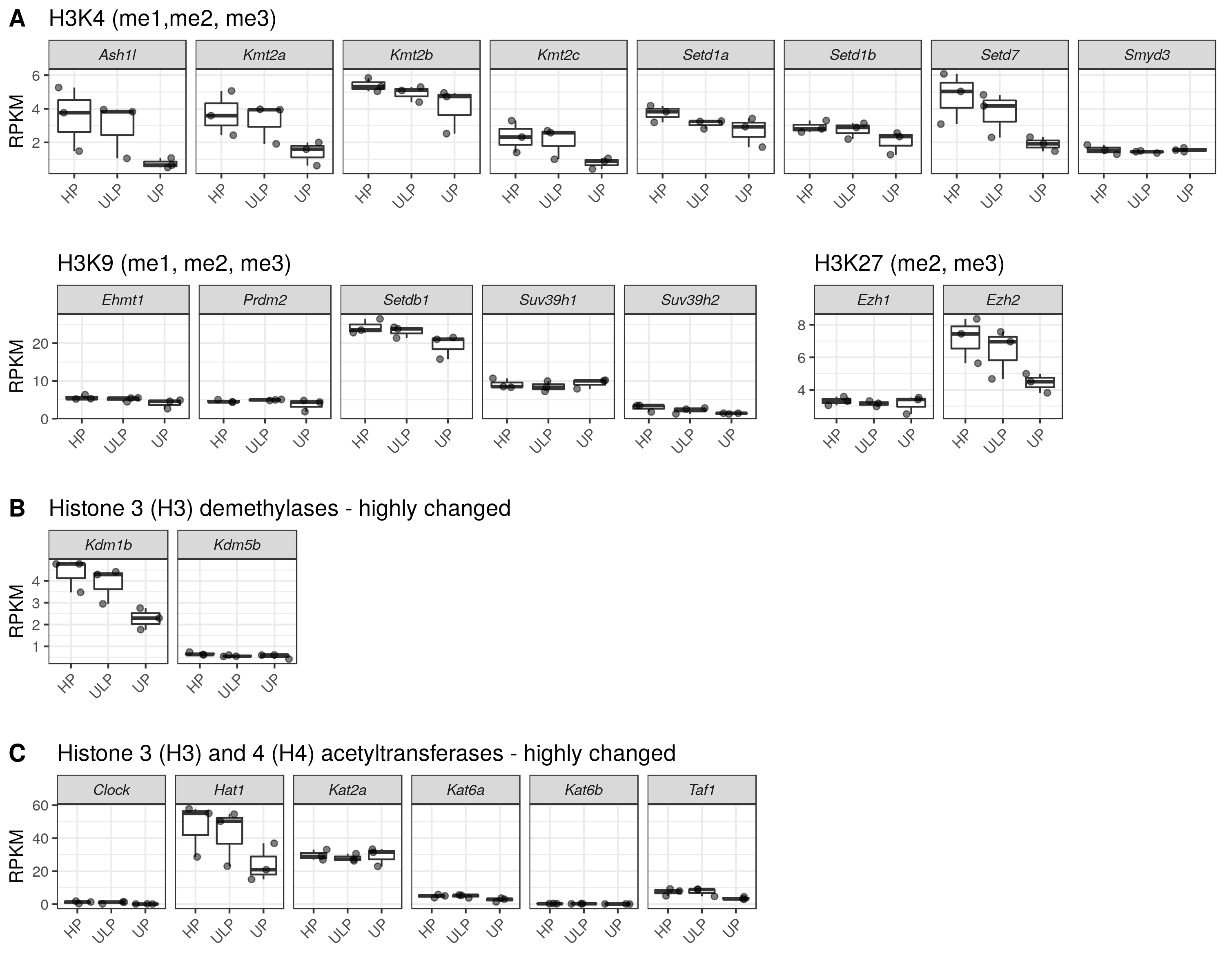


Microglial BV2-cells were stimulated using the two-step approach with fixed priming doses of LPS [ULP (ultra-low dose, 1 fg/mL) and HP (high-dose, 100 ng/mL)] and re-stimulated on day 6 with a fixed dose LPS (100 ng/mL). RNA sequencing were performed in order to characterize expression of genes regulate epigenetic marks on the NFkB pathway. Boxplots show RPKMs of UP, ULP, and HP samples (horizontal line represent the median RPKM value, boxes represent 25-75% quantiles, vertical lines represent 0% and 100% quantile, dots represent RPKMs of individual samples of each group). Of note, both ULP and HP induced distinct increase in methylation and acetylation of histones by A: Mono- (me1), di-(me2) and trimethylation (me3) of histone 3 lysine 4 residues (H3K4), characterized by increased RPKM values of methylation promoters (histone methyltransferases) Ash1l, Kmt2a, Kmt2b, Kmt2c, Setd1a, Setd7 and Setd1b; trimethylation of histone 3 lysine 27 residues (H3K27), characterized by increased RPKM values of methylation promoters (histone methyltransferases) Ezh2. B: Demethylation of histone 3 lysine 9 residues (H3K9) characterized by increased RPKM values of demethylation promoters (histone demethylases) Kdm1b; C: Acetylation of histone 3 (H3) and histone 4 (H4) characterized by increased RPKM values of acetylation promoters (histone demethylases) Hat1; UP, unprimed.

Abbreviations: Ash1l: ASH1 like histone lysine methyltransferase; Kmt2a: lysine (K)-specific methyltransferase 2A; Kmt2a: lysine (K)-specific methyltransferase 2A; Kmt2b: lysine (K)-specific methyltransferase 2B; Kmt2c: lysine (K)-specific methyltransferase 2C; Setd1a: SET Domain Containing 1A, lysine (K)-specific methyltransferase 2F; Setd1b: SET Domain Containing 1B, lysine (K)-specific methyltransferase 2G; Setd7: SET Domain Containing 7, lysine (K)-specific methyltransferase 7; Smyd3: SET and MYND domain containing 3; Ehmt1: euchromatic histone methyltransferase 1; Prdm2: PR domain containing 2, with ZNF domain; Setdb1: SET domain, bifurcated 1; Suv39h1: suppressor of variegation 3-9 homolog 1; Suv39h2: suppressor of variegation 3-9 homolog 2; Ezh1: enhancer of zeste 1 polycomb repressive complex 2 subunit; Ezh2: enhancer of zeste 2 polycomb repressive complex 2 subunit; Kdm1b: lysine (K)-specific demethylase 1B; Kdm5b: lysine (K)-specific demethylase 5B; Clock: circadian locomotor output cycles kaput; Hat1: histone acetyltransferase 1; Kat2a: K(lysine) acetyltransferase 2A; Kat6a: K(lysine) acetyltransferase 6A; Kat6b: K(lysine) acetyltransferase 6B; Taf1: TATA-box binding protein associated factor 1.


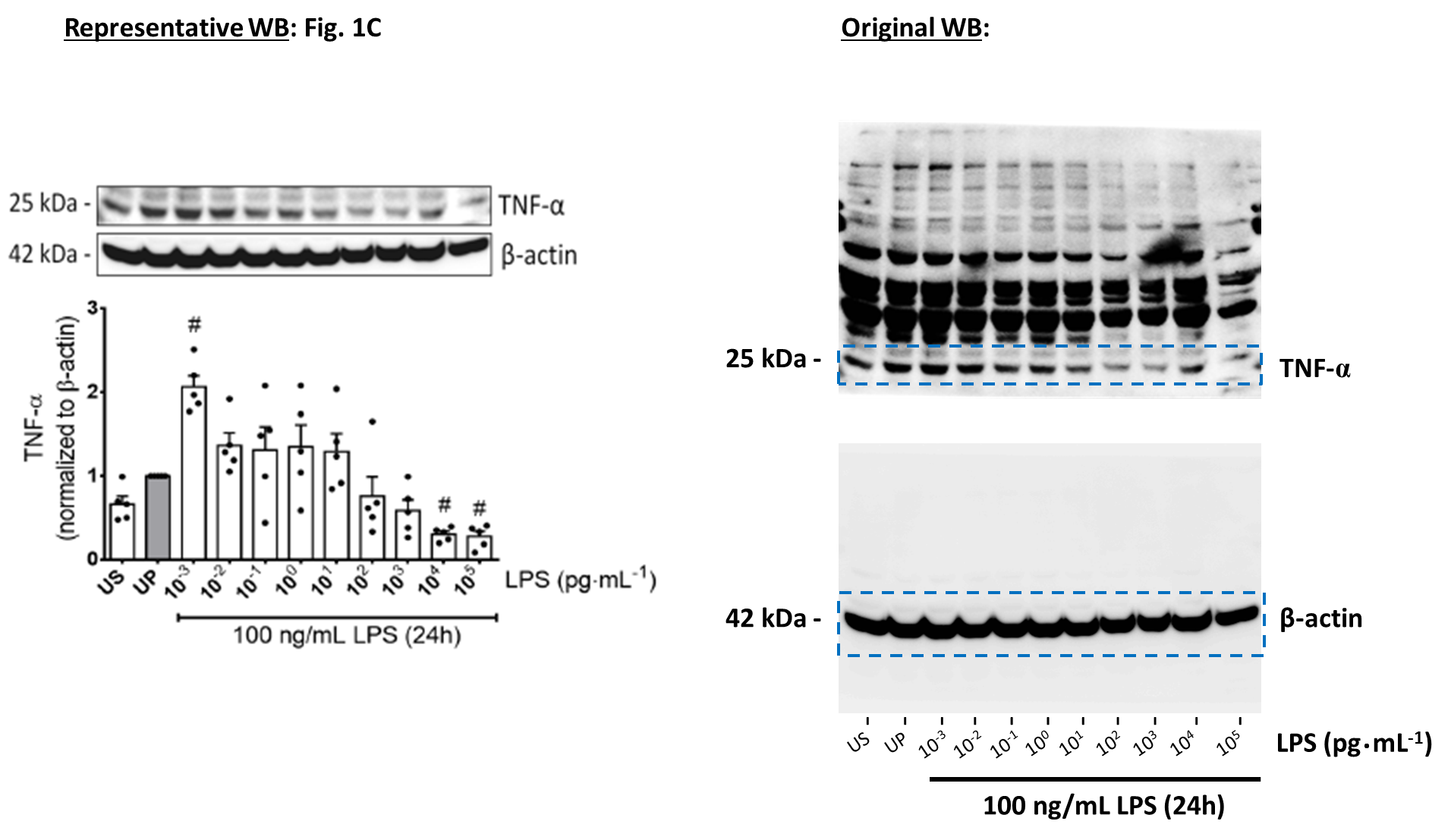


Supplementary Figure 7: **Presentation of the complete western blot (WB) picture of TNF-a content in microglia primed with increasing doses of LPS.** Left panel: Fig. 1C, right panel: Complete western blot. US, unstimulated; UP, unprimed.


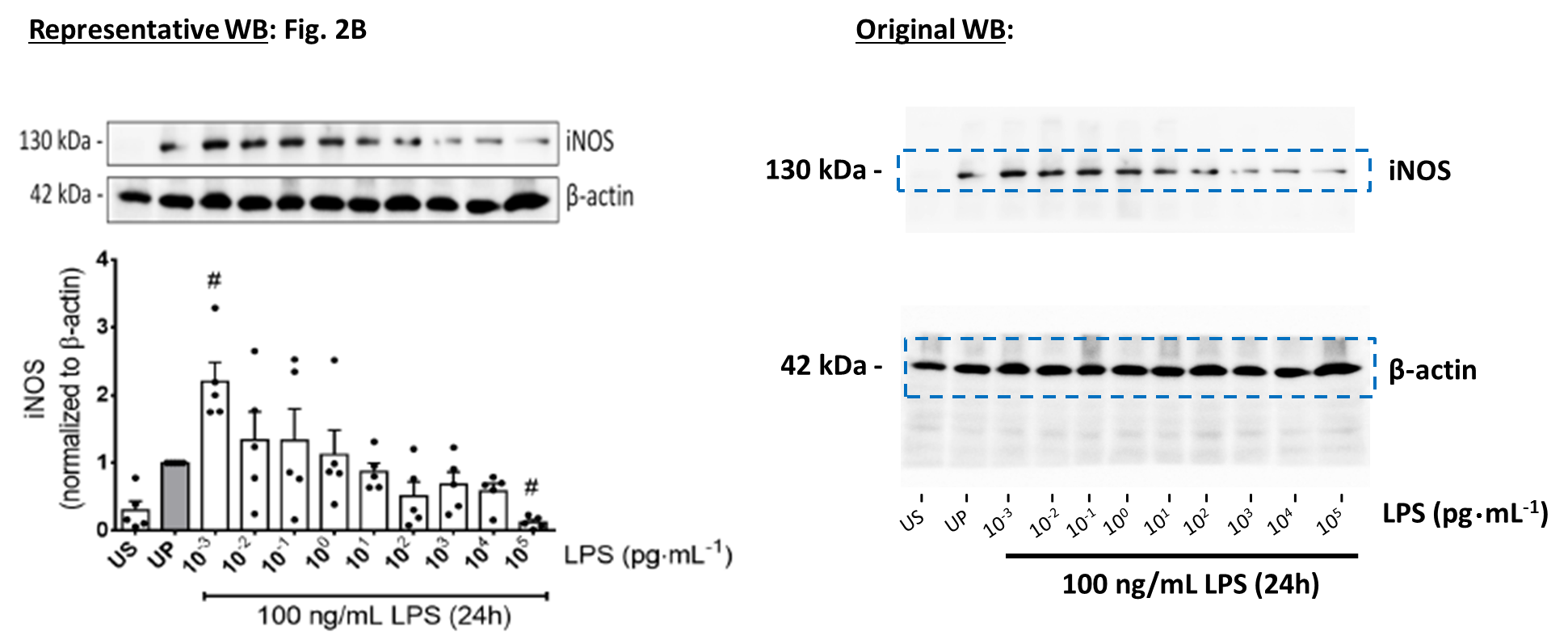


Supplementary Figure 8: **Presentation of the complete western blot (WB) picture of iNOS content in microglia primed with increasing doses of LPS.** Left panel: Fig. 2B, right panel: Complete western blot. US, unstimulated; UP, unprimed.


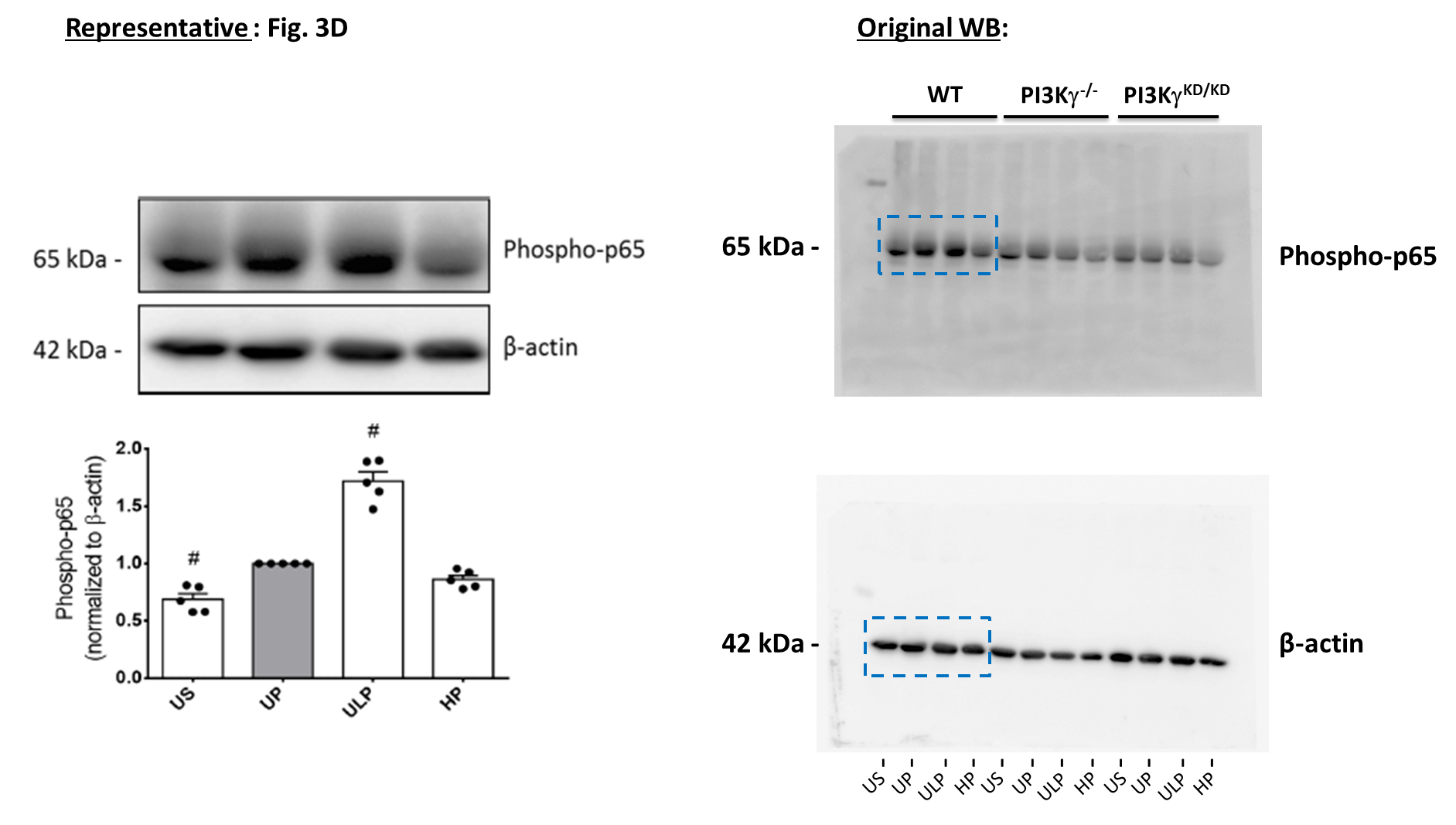


Supplementary Figure 9 **Presentation of the complete western blot (WB) picture of Phospho-p65 content in microglia** primed with a two-step approach with fixed priming doses of LPS [ULP (ultra-low dose, 1 fg/mL) and HP (high-dose, 100 ng/mL)]. Lysates (24h) were collected after the 2nd stimulus with LPS (100 ng/mL)**.** Left panel: Fig. 2B, right panel: Complete western blot. US, unstimulated; UP, unprimed.


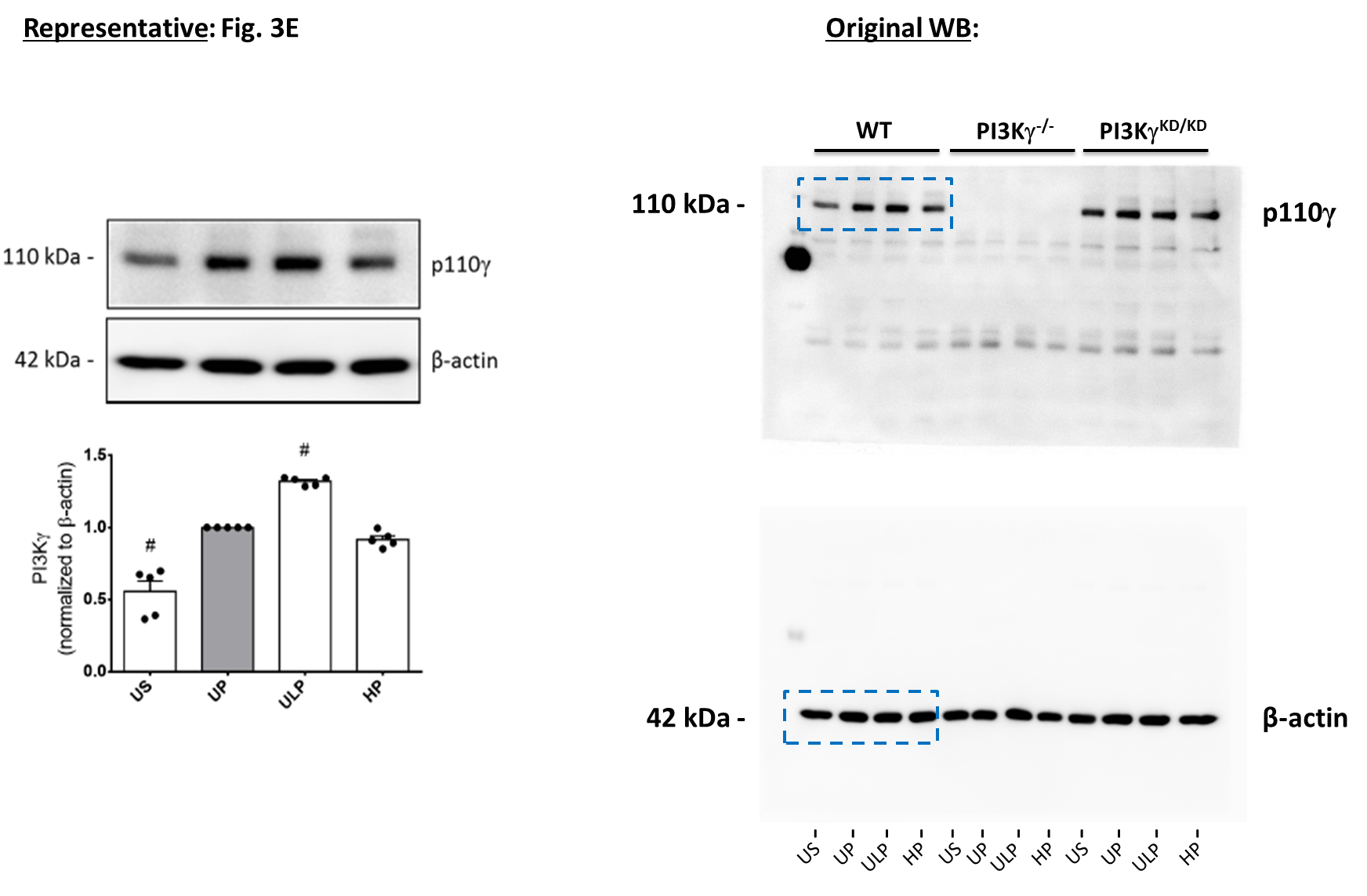


Supplementary Figure 10: **Presentation of the complete western blot (WB) picture of PI3Kγ subunit p110γ content in microglia** primed with two-step approach with fixed priming doses of LPS [ULP (ultra-low dose, 1 fg/mL) and HP (high-dose, 100 ng/mL)]. Lysates (24h) were collected after the 2nd stimulus with LPS (100 ng/mL). Left panel: Fig. 3E, right panel: Complete western blot. US, unstimulated; UP, unprimed.


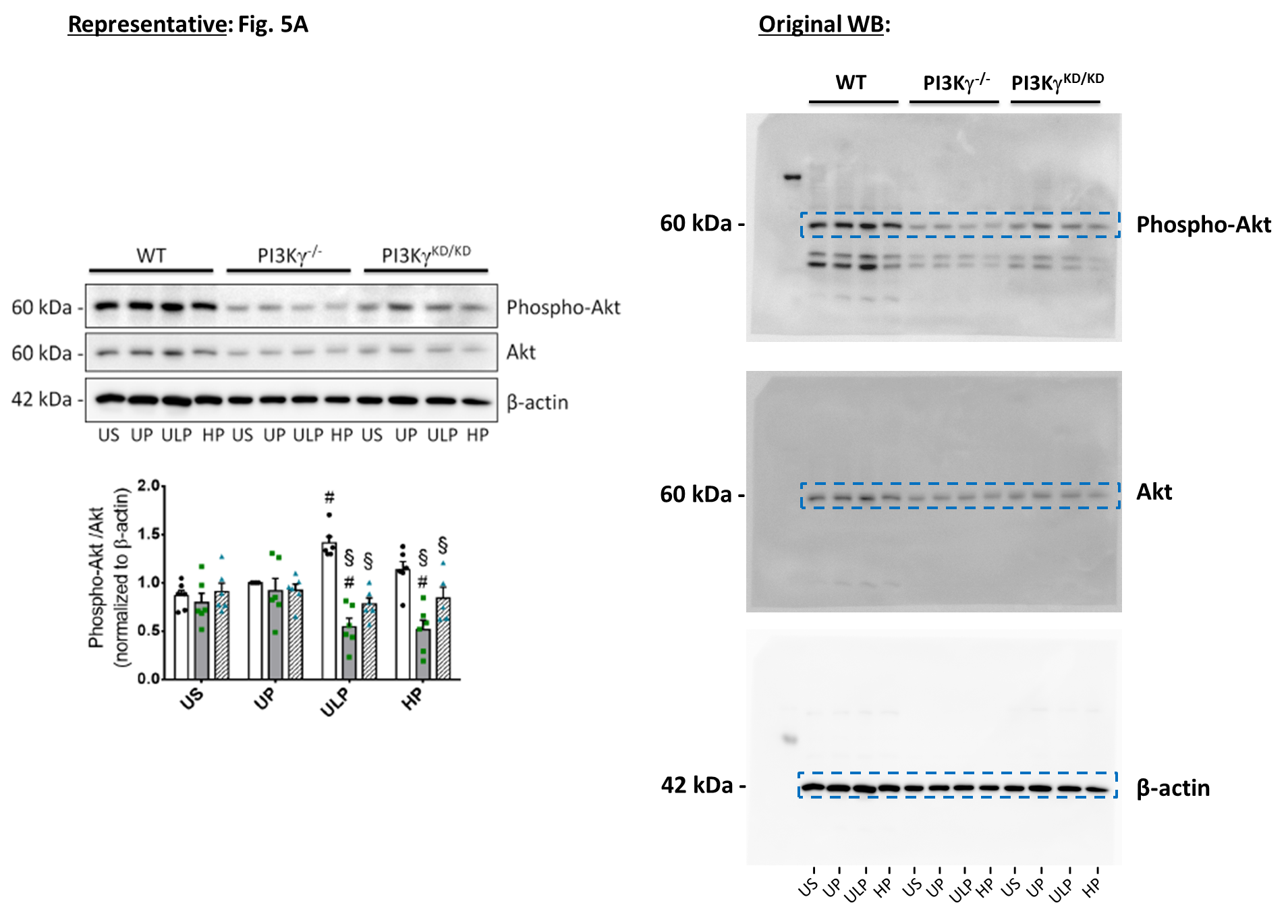


Supplementary Figure 11: **Presentation of the complete western blot (WB) picture of Phospho-Akt content in microglia** primed with two-step approach with fixed priming doses of LPS [ULP (ultra-low dose, 1 fg/mL) and HP (high-dose, 100 ng/mL)]. Lysates (24h) were collected after the 2nd stimulus with LPS (100 ng/mL)**.** Left panel: Fig. 5A, right panel: Complete western blot. US, unstimulated; UP, unprimed.


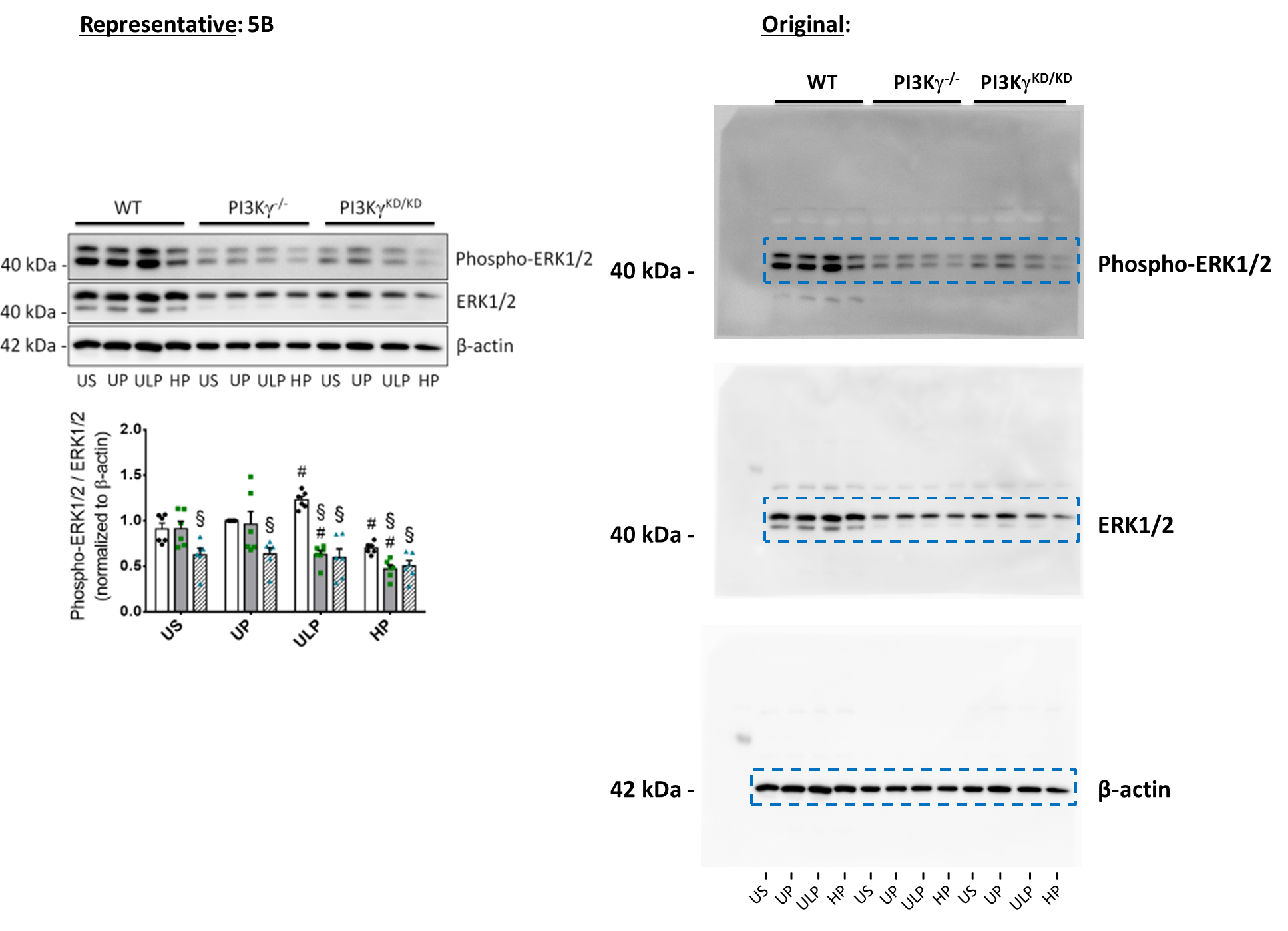


Supplementary Figure 12: **Presentation of the complete western blot (WB) picture of Phospho-ERK1/2 content in microglia** primed with two-step approach with fixed priming doses of LPS [ULP (ultra-low dose, 1 fg/mL) and HP (high-dose, 100 ng/mL)]. Lysates (24h) were collected after the 2nd stimulus with LPS (100 ng/mL)**.** Left panel: Fig. 5B, right panel: Complete western blot. US, unstimulated; UP, unprimed


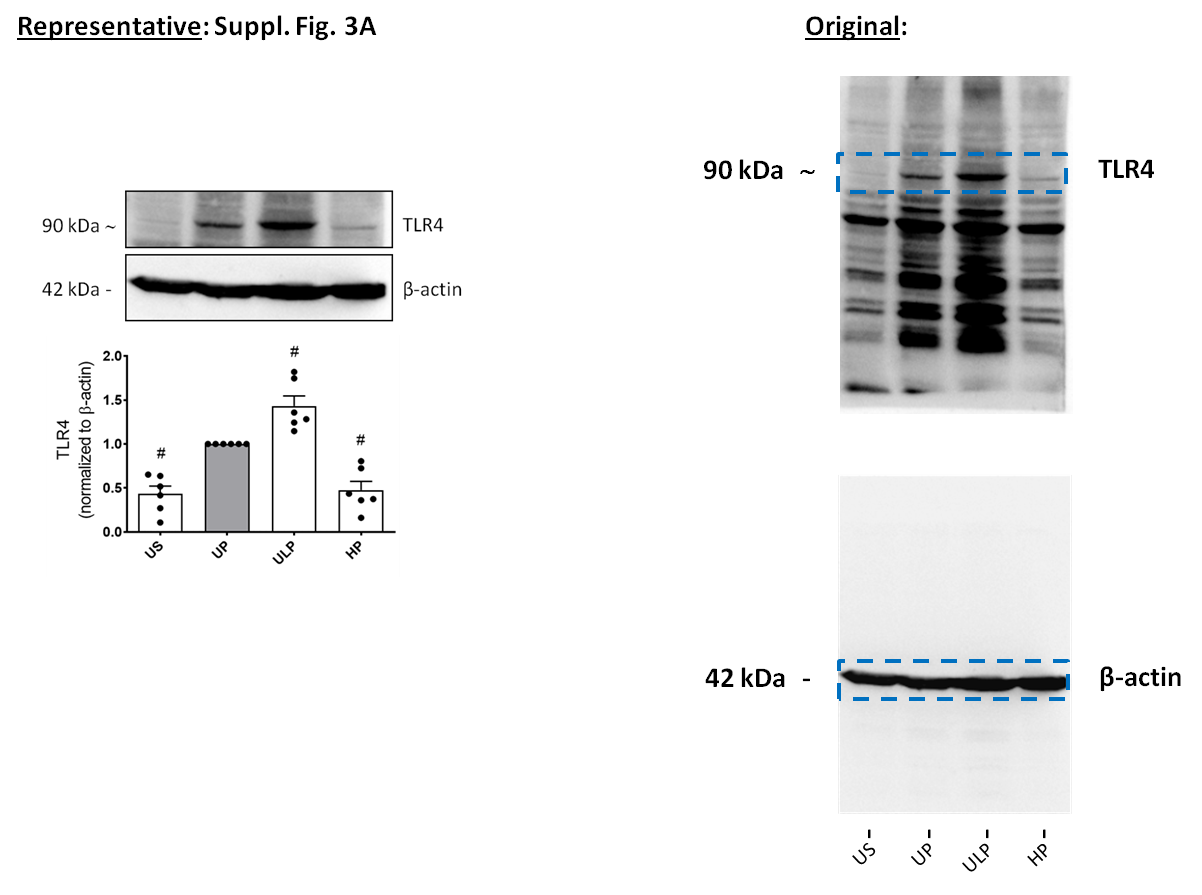


Supplementary Figure 13: **Presentation of the complete western blot (WB) picture of TLR4 content in microglia** primed with a two-step approach with fixed priming doses of LPS [ULP (ultra-low dose, 1 fg/mL) and HP (high-dose, 100 ng/mL)]. Lysates (24h) were collected after the 2nd stimulus with LPS (100 ng/mL)**.** Left panel: Fig. 3A, right panel: Complete western blot. US, unstimulated; UP, unprimed.


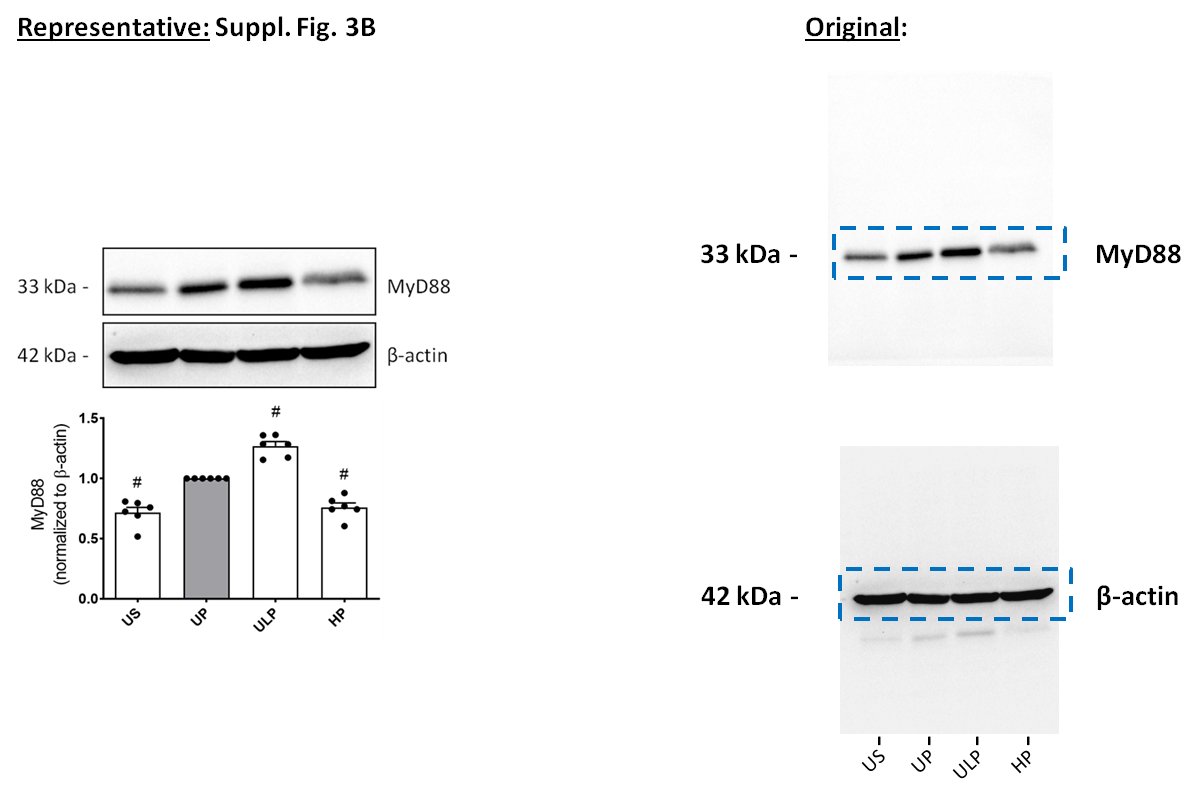


Supplementary Figure 14: **Presentation of the complete western blot (WB) picture of MyD88 content in microglia** primed with two-step approach with fixed priming doses of LPS [ULP (ultra-low dose, 1 fg/mL) and HP (high-dose, 100 ng/mL)]. Lysates (24h) were collected after the 2nd stimulus with LPS (100 ng/mL)**.** Left panel: Suppl. Fig. 3B, right panel: Complete western blot. US, unstimulated; UP, unprimed.

**References**

Bentley, D.R., Balasubramanian, S., Swerdlow, H.P., Smith, G.P., Milton, J., Brown, C.G., Hall, K.P., Evers, D.J., Barnes, C.L., Bignell, H.R., Boutell, J.M., Bryant, J., Carter, R.J., Keira Cheetham, R., Cox, A.J., Ellis, D.J., Flatbush, M.R., Gormley, N.A., Humphray, S.J., Irving, L.J., Karbelashvili, M.S., Kirk, S.M., Li, H., Liu, X., Maisinger, K.S., Murray, L.J., Obradovic, B., Ost, T., Parkinson, M.L., Pratt, M.R., Rasolonjatovo, I.M., Reed, M.T., Rigatti, R., Rodighiero, C., Ross, M.T., Sabot, A., Sankar, S.V., Scally, A., Schroth, G.P., Smith, M.E., Smith, V.P., Spiridou, A., Torrance, P.E., Tzonev, S.S., Vermaas, E.H., Walter, K., Wu, X., Zhang, L., Alam, M.D., Anastasi, C., Aniebo, I.C., Bailey, D.M., Bancarz, I.R., Banerjee, S., Barbour, S.G., Baybayan, P.A., Benoit, V.A., Benson, K.F., Bevis, C., Black, P.J., Boodhun, A., Brennan, J.S., Bridgham, J.A., Brown, R.C., Brown, A.A., Buermann, D.H., Bundu, A.A., Burrows, J.C., Carter, N.P., Castillo, N., Chiara, E.C.M., Chang, S., Neil Cooley, R., Crake, N.R., Dada, O.O., Diakoumakos, K.D., Dominguez-Fernandez, B., Earnshaw, D.J., Egbujor, U.C., Elmore, D.W., Etchin, S.S., Ewan, M.R., Fedurco, M., Fraser, L.J., Fuentes Fajardo, K.V., Scott Furey, W., George, D., Gietzen, K.J., Goddard, C.P., Golda, G.S., Granieri, P.A., Green, D.E., Gustafson, D.L., Hansen, N.F., Harnish, K., Haudenschild, C.D., Heyer, N.I., Hims, M.M., Ho, J.T., Horgan, A.M., et al. (2008). Accurate whole human genome sequencing using reversible terminator chemistry. *Nature* 456**,** 53-59.

Kim, D., Pertea, G., Trapnell, C., Pimentel, H., Kelley, R., and Salzberg, S.L. (2013). TopHat2: accurate alignment of transcriptomes in the presence of insertions, deletions and gene fusions. *Genome Biol* 14**,** R36.

Liao, Y., Smyth, G.K., and Shi, W. (2014). featureCounts: an efficient general purpose program for assigning sequence reads to genomic features. *Bioinformatics* 30**,** 923-930.

Team, R.C. (2019). "R: A language and environment for statistical computing". (Vienna, Austria: R Foundation for Statistical Computing. URL: <https://www.R-project.org/>).

Zerbino, D.R., Achuthan, P., Akanni, W., Amode, M.R., Barrell, D., Bhai, J., Billis, K., Cummins, C., Gall, A., Giron, C.G., Gil, L., Gordon, L., Haggerty, L., Haskell, E., Hourlier, T., Izuogu, O.G., Janacek, S.H., Juettemann, T., To, J.K., Laird, M.R., Lavidas, I., Liu, Z., Loveland, J.E., Maurel, T., Mclaren, W., Moore, B., Mudge, J., Murphy, D.N., Newman, V., Nuhn, M., Ogeh, D., Ong, C.K., Parker, A., Patricio, M., Riat, H.S., Schuilenburg, H., Sheppard, D., Sparrow, H., Taylor, K., Thormann, A., Vullo, A., Walts, B., Zadissa, A., Frankish, A., Hunt, S.E., Kostadima, M., Langridge, N., Martin, F.J., Muffato, M., Perry, E., Ruffier, M., Staines, D.M., Trevanion, S.J., Aken, B.L., Cunningham, F., Yates, A., and Flicek, P. (2018). Ensembl 2018. *Nucleic Acids Res* 46**,** D754-D761.
